# Global trends of laser bone ablation: bibliometric analysis of publications from 1979 to 2023

**DOI:** 10.3389/fsurg.2025.1461319

**Published:** 2025-03-11

**Authors:** Kai-Jun Zhang, Qi Liu, Teng Zhang, Anjie Shen, Wei Han, Jinqi Li, Bin Zhao, Junqiang Wang

**Affiliations:** ^1^ Peking University Fourth School of Clinical Medicine, Beijing, China; ^2^ Department of Orthopaedic Trauma, Beijing Jishuitan Hospital, Capital Medical University, Beijing, China; ^3^ Seventh Clinical Medical College of Capital Medical University, Beijing, China; ^4^ Beijing Jishuitan Orthopaedic Robot Engineering Research Center Co., Ltd., Beijing, China

**Keywords:** laser, bone ablation, bibliometrics analysis, visual analysis, CiteSpace, VOSviewer

## Abstract

**Objectives:**

The objective of the bibliometric analysis was to quantify and identify the current status and trends of laser bone ablation research from 1979 to 2023.

**Materials and methods:**

The Web of Science (WOS) core collection database was used to search for articles on laser bone ablation published from 1979 to 2023. The collected data were then imported into Microsoft Excel, VOSviewer, and CiteSpace for detailed analysis and visualization.

**Results:**

A total of 383 articles were included for analysis. The United States made the most significant contributions to the field in terms of both quantity and quality. Moreover, Cattin, Philippe C emerged as the author with the highest number of publications, while the University of Basel stood out as the institution with the greatest publication output. *Lasers in Surgery and Medicine* emerged as not only the journal with the most publications but also held considerable influence within its domain. Prominent keywords that surfaced frequently included “ablation,” “er:yag laser,” and “bone.”

**Conclusion:**

The annual number of publications in the field of laser bone ablation is showing an overall upward trend. Research on laser bone ablation primarily focuses on investigating the parameters of this technique, as well as its application in treating bone tumors, performing laser stapes surgery, and various applications of laser bone ablation. The laser osteotomy, laser ablation of bone tumors, animal experiments, and the interaction with biological tissues during laser bone ablation are expected to be the focal areas and future directions in this field.

## 1 Introduction

Since the emergence of the first practical laser in the 1960s (1), lasers have gained widespread utilization within the medical field (2). Laser therapy offers numerous advantages, including infection risk reduction, hemorrhage mitigation, accelerated wound healing, and enhanced surgical approach (3). In recent years, there has been a growing focus on employing lasers in orthopedics, maxillofacial surgery, otologic surgery, and other surgical disciplines (4).

At present, traditional mechanical instruments, such as pendulum saws and drills, are still the standard tools for orthopedic surgery. However, these devices require direct contact with bone to function, resulting in heat, pressure, vibration, and noise (5). The heat generated by mechanical tools can lead to the damage of bone cells and even osteonecrosis, thus weakening the contact between the internal fixator and bone, loosening and failure of internal fixator, and eventually leading to delayed fracture union or even non-union of fractures (6). Both the pressure exerted by the surgeon on the bone surface during the use of traditional tools and the vibration caused by mechanical operation can cause new damage to the bone, and even lead to bone fragmentation (7, 8). Mechanical devices tend to generate louder noise than lasers (9, 10). So the discomfort for the patient and the surgeon is often even greater.

Laser ablation has many advantages over traditional instruments, thus it has gradually gained acceptance and implementation in clinical practice for bone tissue (11, 12). Laser ablation of bone tissue is a non-contact operation with almost no pressure and vibration, and less noise and thermal damage (13). The non-contact operation can prevent pathogens from entering the incision through the instrument to reduce wound infection (3). Unlike mechanical tools, laser ablation allows for cutting complex geometric shapes without being limited by tip shape (14). In addition, laser ablation has higher accuracy, which can reduce the risk of damage to the tissue around the bone. The integration of computer-assisted and robot-guided ablation can enhance the precision and efficiency of ablation, thereby presenting promising prospects for further development (15). Animal experiments have proved the advantages of laser bone ablation over traditional mechanical methods. Lo et al. (16) produced skull defects in mice with trephine drill bit or femtosecond laser, respectively, and observed that wound healing in the laser group was significantly faster than that in the mechanical group at 2, 4, and 6 weeks after surgery. Gabriolic et al. (17) drilled the pig ribs with Er:YAG laser and surgical drill respectively, and found that the cavity prepared by the laser was regular with clear and sharp edges, and the edge of the specimen prepared by the electric drill group was irregular, and the time spent by the laser group was significantly shorter than that of the drilling group.

It is imperative to facilitate a comprehensive understanding of the current advancements and focal points in laser bone ablation for researchers. Bibliometrics, as a research methodology, offers insights into the characteristics and progress within a specific subject area (18). It is frequently combined with visual information to find connections between institutions, journals, countries, and identify emerging research trends (19). To our knowledge, no studies have conducted a bibliometric analysis on laser bone ablation. The objectives of this study were: (1) to analyze keywords, references, journals, authors, institutions, and countries using bibliometric analysis in order to uncover the research characteristics of laser bone ablation; (2) to visualize data that reflects interconnections among different authors, institutions, countries, and journals, with the aim of exploring prominent areas of research interest and prevailing trends.

## 2 Materials and methods

### 2.1 Data sources

The Web of Science Core Collection database (WOSCC) was utilized for a search conducted on October 21, 2023.

### 2.2 Search strategy

The search terms were TS = (“laser” OR “lasers”) AND TS = (“Bone” OR “Bones” OR “Bone and Bones” AND “Condyle” OR “Condyles” OR “Femur” OR “Femurs” OR “tibia” OR “tibias” OR “tibiae” OR “Mandibles” OR “Mandible” OR “Maxilla” OR “Maxillas” OR “Maxillae” OR “Maxillary” OR “maxillaires” OR “Frontal Bone” OR “Frontal Bones” OR “Parietal Bone” OR “Parietal Bones” OR “Basilar Bone” OR “Basilar Bones” OR “Occipital Bone” OR “Occipital Bones” OR “sternum” OR “sternum” OR “sterna” OR “Stapes” OR “stage” OR “stage bone” OR “stage bones”) AND TS = (“cut” OR “drill*”OR “bur” OR “burs” OR “burr” OR “burrs” OR “ablation” OR “ablate” OR “osteotomy” OR “osteotomies” OR “osteotomies” OR “osteotome”) NOT TS = (“Laser Scans” OR “laser scanner” OR “laser scanning” OR “laser-scanning” OR “laser-Doppler” OR “laser Doppler” OR “laser guidance” OR “laser navigation” OR “laser angiography” OR “laser sintering” OR “laser printing” OR “laser microscopy”). To avoid potential bias, the language type of publications was limited to English, and only reviews and articles were included.

### 2.3 Inclusion and exclusion criteria

The inclusion criteria of publication were: (a) The themes included ablation or drilling or cutting of bone using lasers. The exclusion criteria were as follows: (a) The themes were laser ablation or drilling or cutting of the cartilage; (b) The themes were ablation or drilling or cutting of dental tissue; (c) The methodology included laser ablation-inductively coupled plasma-mass spectrometry (LA-ICP-MS), Laser ablation U-series and laser-induced breakdown spectroscopy (LIBS) either individually or in combination, but not applied to laser ablation or drilling or cutting of bone tissue.

### 2.4 Data collection

The complete records were extracted from the publications retrieved by two independent authors (ZKJ and LQ). Disagreements were resolved through discussion to mitigate potential bias. The acquired publication information was exported in TXT format.

### 2.5 Data analysis

The bibliometric indicators utilized in this study encompassed the total publications, total times cited, average citations per item, H-index, and self-citation times. The total publications are widely employed as a metric to gauge contribution within a specific field. The total times cited and the average citations per item indicate the level of attention (20). The H-index serves as an indicator for both the quantity and quality of an author's published papers; it signifies that an academic has published H papers, each receiving at least H citations from other publications. Furthermore, it can also be used to assess the publication quality of countries/regions, institutions, or journals (21, 22).

In this study, Microsoft Excel 2016 was employed for publication data analysis and graphics. VOSviewer (V.1.6.18) was utilized to visually analyze countries, authors, institutions, journals, and keywords. In graphs drawn using VOSviewer, items are usually formed by circles and labels. The Total Link Strength (TLS) served as a bibliometric indicator to measure the influence and collaboration of items. Furthermore, The CiteSpace software was employed to analyze citation burst the dual-map of journals, and the keyword timelines. The parameters used were as follows: time span (1979–2022), years per slice (1), scale factor *k* = 25, and selection criteria (top *N* = 50). Cluster labels were extracted using the Log-Likelihood Ratio (LLR) algorithm while other parameter settings remained consistent with the initial software configuration.

## 3 Results

### 3.1 Analysis of article numbers and trends

**Figure 1:**
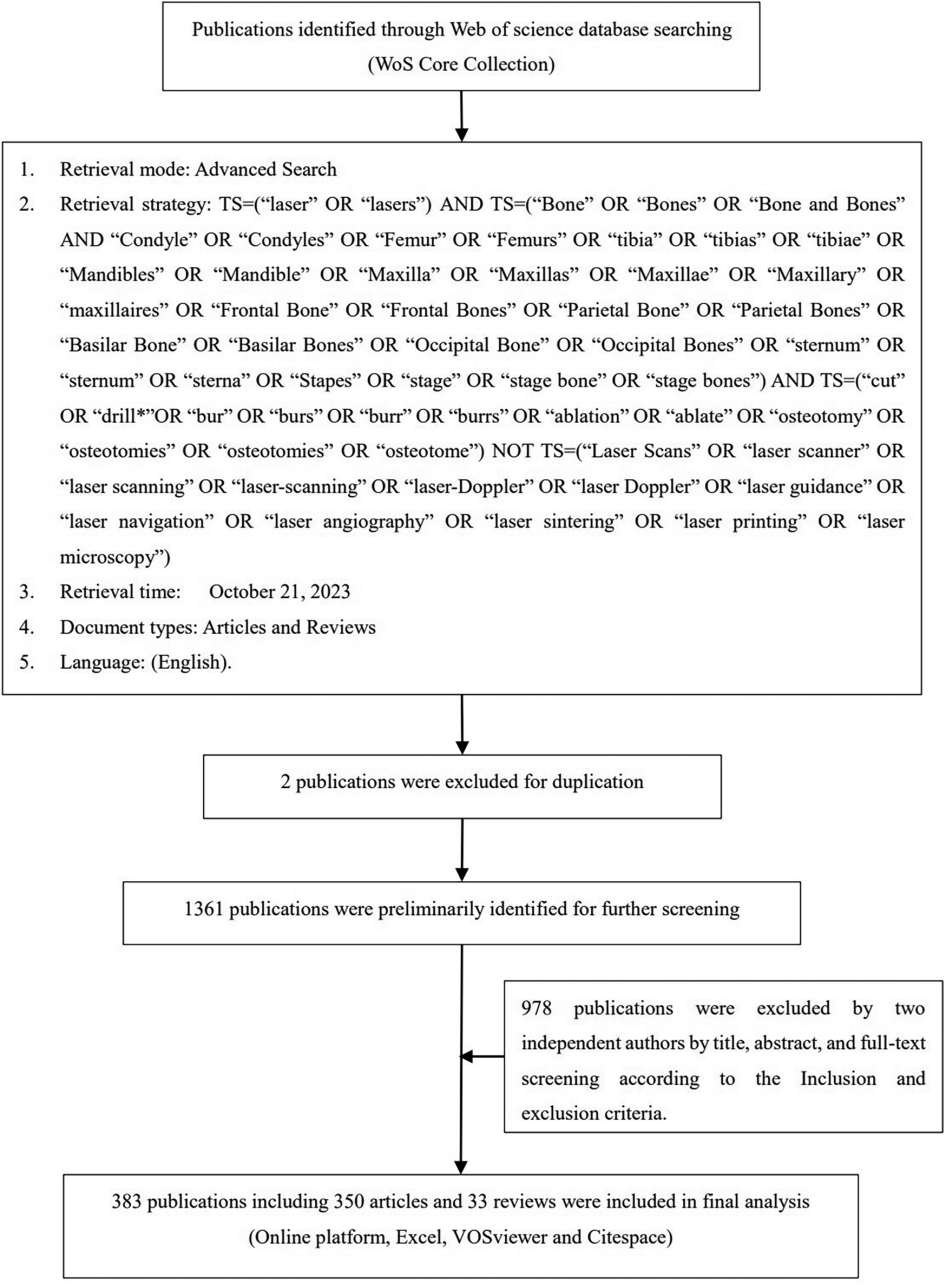
Flow diagram of the inclusion process in bibliometric analysis of laser bone ablation.

According to the search strategy, a total of 1,363 publications were retrieved, and then two independent researchers (ZKJ and LQ) manually reviewed the titles, abstracts, and full texts of the publications according to the inclusion and exclusion criteria. Finally, 383 publications were included, consisting of 350 articles and 33 reviews, published from 1979 to 2023. The detailed publication search and selection process is shown in Figure 1.

**Figure 2:**
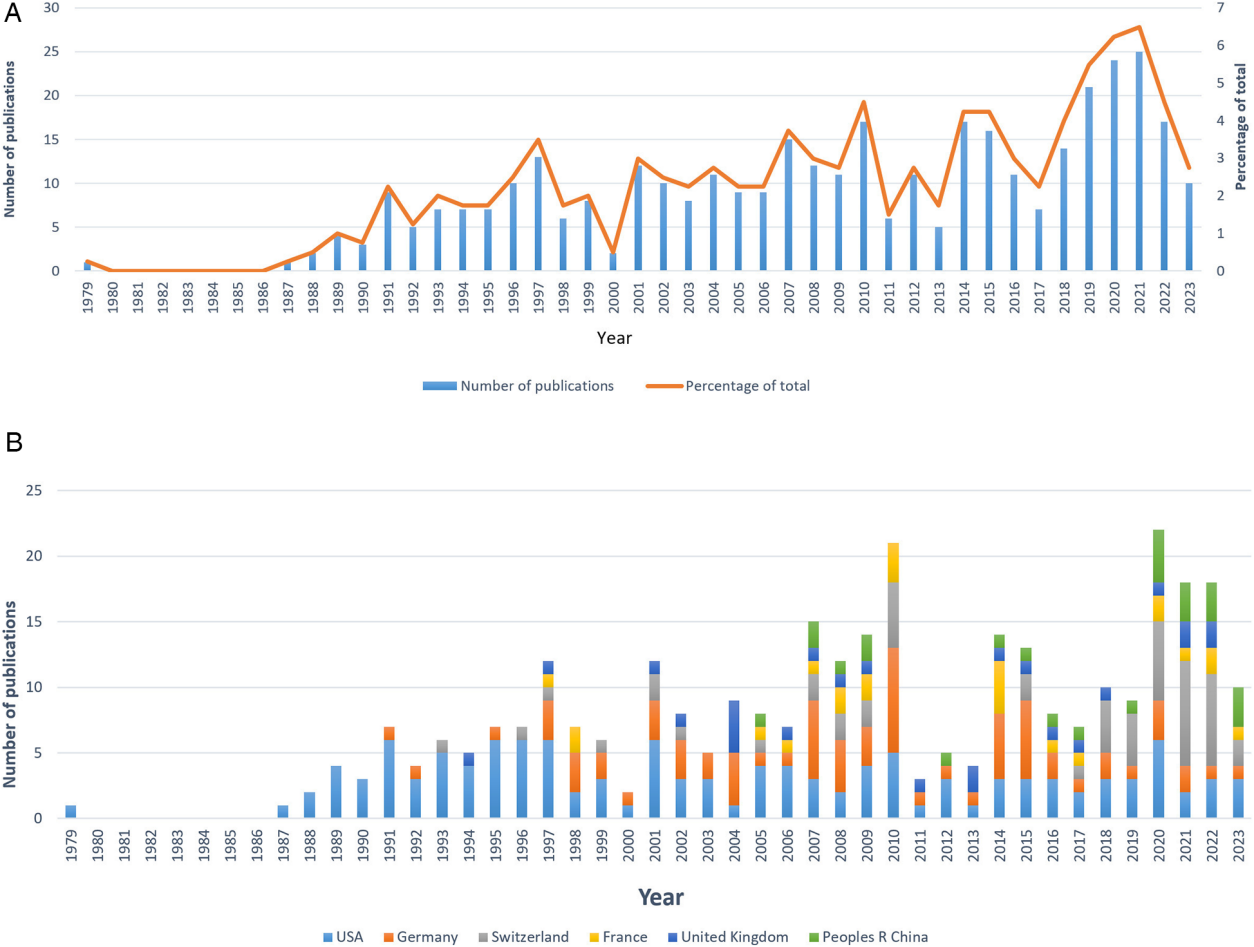
Global trends of publications on laser bone ablation from 1979 to 2023. **(A)** Annual trends in the publications worldwide. **(B)** Publications of the top six countries over time.

As shown in Figure 2A, the annual number of publications in this field shows an overall upward trend, characterized by intermittent fluctuations, from 1996 to 2019. Before 1996, the number of publications was at a low level, and the annual number of publications has not exceeded two digits. From 2019 to 2021, the number of articles published each year was more than 20, and the number of articles published in 3 years accounted for 19.9% of the total number of articles published. Notably, the highest number of publications was observed in 2021, reaching a remarkable count of 25. The number of publications in 2022 was lower than that in 2019–2021.

### 3.2 Analysis of nations

According to the bibliometric analysis, a total of 44 countries/regions have published articles on laser bone ablation from 1979 to 2023. The top 10 countries in terms of total publications are already listed in Supplementary Table 1. The United States has made the highest contribution (*n* = 124/32.38%), followed by Germany (*n* = 74/19.32%), Switzerland (*n* = 53/13.84%), France (*n* = 25/6.53%), the United Kingdom (*n* = 25/6.53%), and the People's Republic of China (*n* = 25/6.53%).

**Figure 3:**
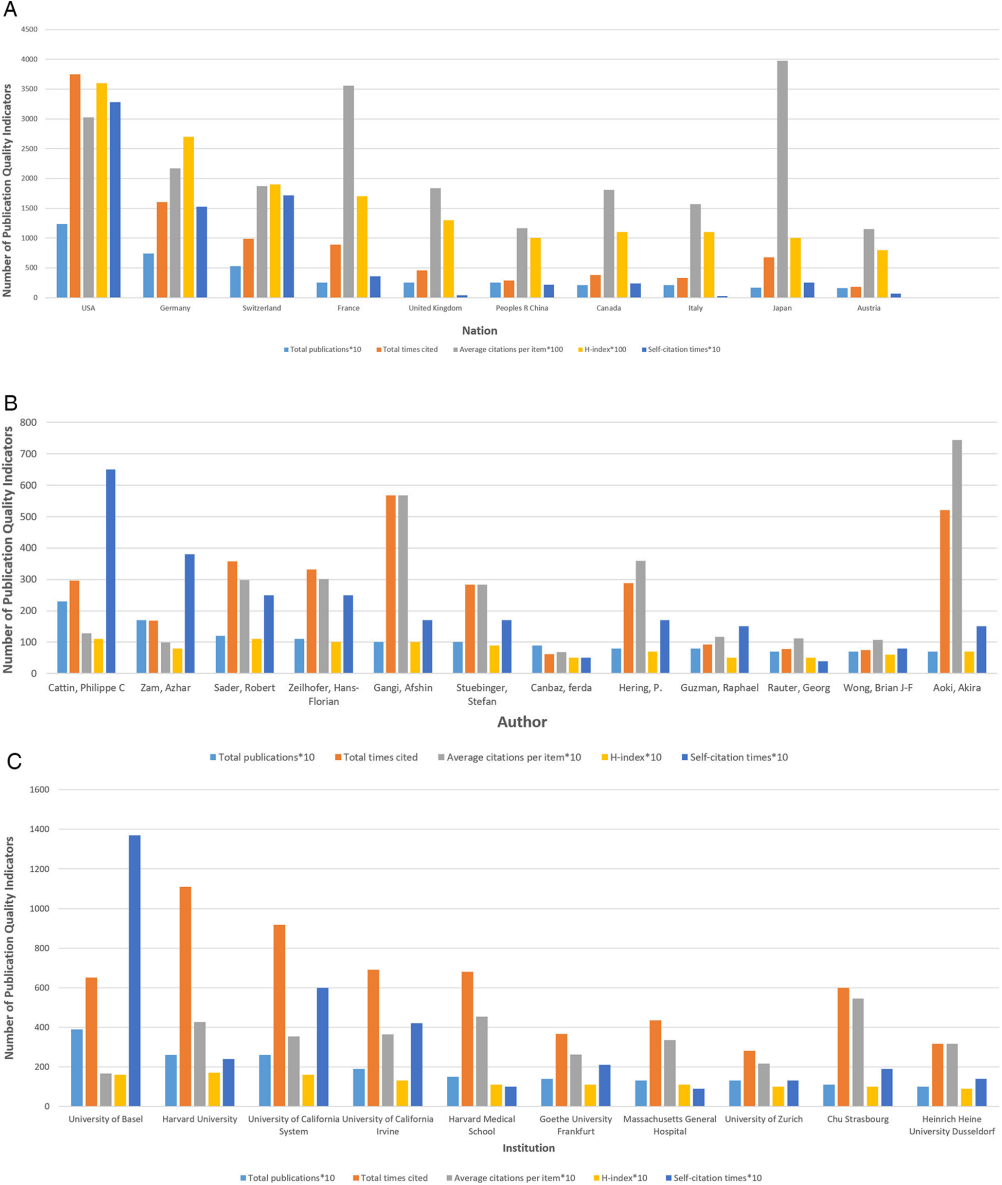
Quality analysis of global publications on laser bone ablation from 1979 to 2023. **(A)** Total publications, total times cited, average citations per item, H-index, and self-citation times of the top ten countries by contributions. **(B)** Total publications, total times cited, average citations per item, H-index, and self-citation times of the top 12 authors by contributions. **(C)** Total publications, total times cited, average citations per item, H-index, and self-citation times of the top 10 institutions by contributions.

Figure 3A presents the publication quality indicators for the top ten countries in the total publications, including total times cited, average citations per item, the H-index, and the self-citation times. Among the 10 countries, the United States ranked first in total times cited (3,751), H-index (36), and self-citation times (328) compared with the other nine countries, but ranked third in average citations per item (30.25). Germany ranked second in the total times cited (1,606) and H-index (27), but ranked third in the self-citation times (153) and fourth in the average citations per item (21.70). The average citations per item of other countries were as follows: Australia (11.50), the People's Republic of China (11.64), Italy (15.71), Canada (18.10), the United Kingdom (18.36), Switzerland (18.72), France (35.56) and Japan (39.76), with Japan ranking first. In addition, the two countries with the lowest self-citation times were Italy (3) and the United Kingdom (4). According to Figure 2B, among the top six countries in terms of total publications, the United States published the first article in this field in 1979.

**Figure 4:**
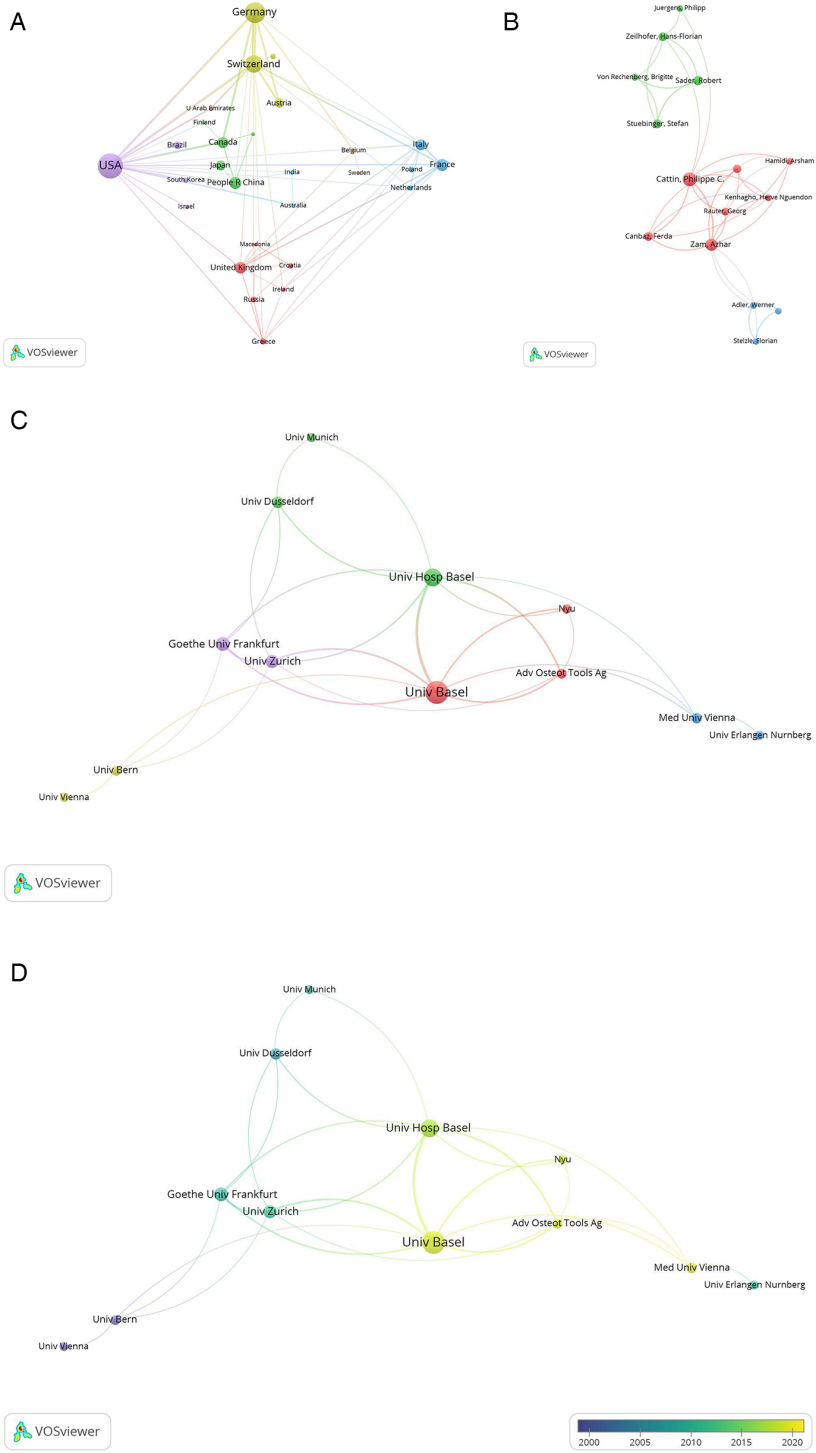
Co-authorship analysis of global publications on laser bone ablation from 1979 to 2023. **(A)** Network visualization map of the 31 countries identified in the laser bone ablation research. **(B)** Network visualization map of the top 31 authors identified in the laser bone ablation research. **(C)** Network visualization map of the 26 institutions involved in the laser bone ablation research. In the Network visualization map, circles and labels form items, and the circle size represents the number of total publications. The thickness of the lines represents the correlation strength between the items, and the color of the circles represents different clusters. **(D)** Overlay visualization map of the 26 institutions involved in the laser bone ablation research. In the overlay visualization map, the color of the Node represents the average year of publication of the literature. The bluer the node, the earlier the document was published; the yellower the color of the node, the later the document was published.

Figure 4A is a Network visualization map of co-authorship analysis of countries participating in this research field, in which the circles and labels form items, and the circle size represents the number of total publications. The thickness of the lines represents the strength of the correlation between the items, and the color of the circles represents different clusters. Co-authorship analysis is a method of assessing the strength of collaboration between items by counting the number of co-authored publications (23). The minimum number of publications per country was set at two, and 31 countries met the requirement. In the co-authorship analysis graph, the TLS represents the degree of item collaboration, and the three countries with the highest TLS are the United States (*n* = 49), Switzerland (*n* = 43), and Germany (*n* = 38).

**Figure 5:**
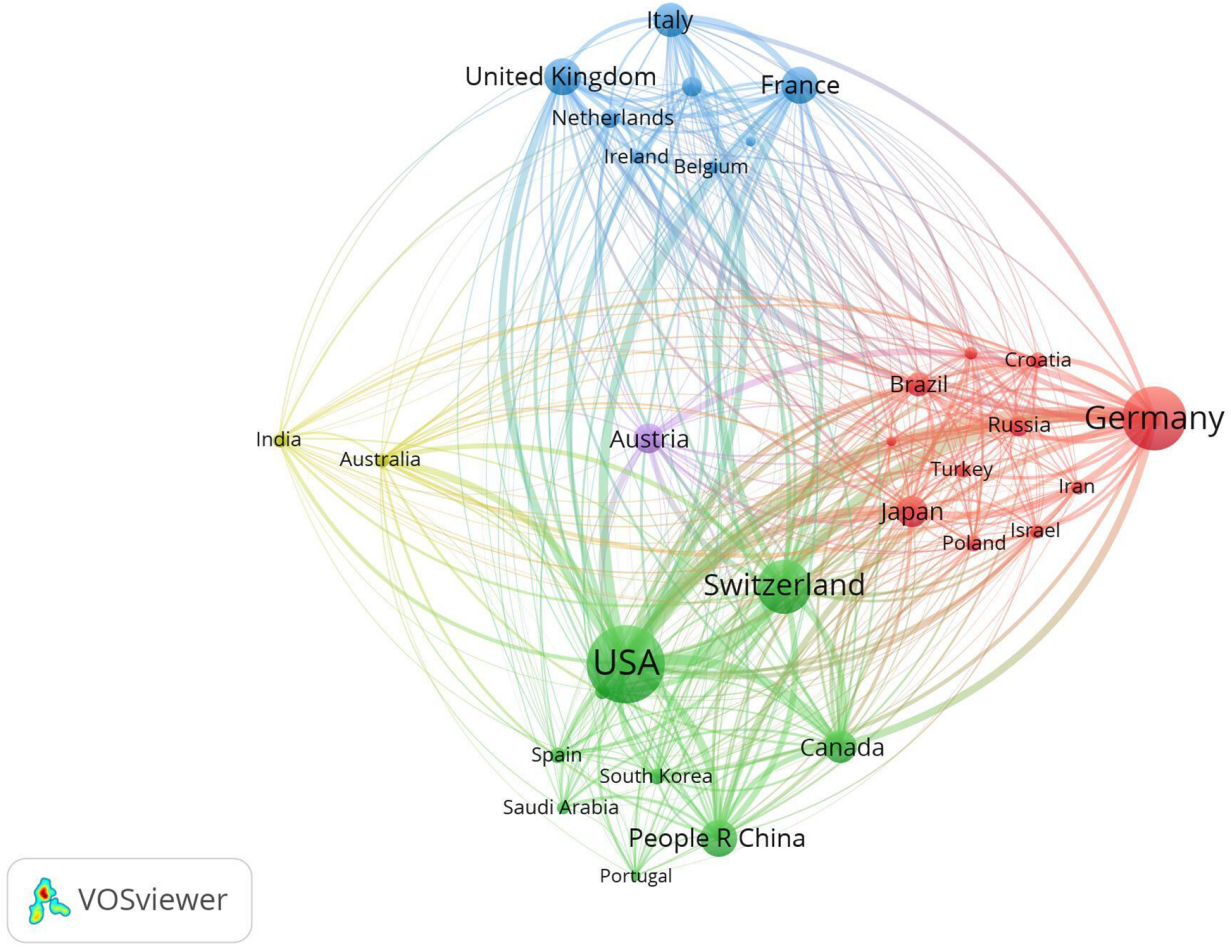
Network visualization map of bibliographic coupling analysis of the 31 countries identified in the laser bone ablation research. In the Network visualization map, circles and labels form items, and the circle size represents the number of total publications. The thickness of the lines represents the correlation strength between the items, and the color of the circles represents different clusters.

Figure 5 is a Network visualization map of bibliographic coupling analysis of countries participating in this research field. Bibliographic coupling analysis is a way to show similar relationships between items via the number of references co-cited by items. The minimum number of publications per country was set at two, and 31 countries met the requirement. In this bibliographic coupling analysis graph, the TLS represents the power of the country in this research field (24), and the three countries with the highest TLS are the United States (*n* = 21,228), Germany (*n* = 14,501), and Switzerland (*n* = 13,567). It indicated that the United States, Germany, and Switzerland were regarded as the leading countries in the research of laser bone ablation.

### 3.3 Analysis of authors

The authors who made the most significant contributions to this area of research are listed in Supplementary Table 2 and Figure 3B. These 12 authors collectively published a total of 66 articles, accounting for approximately 17.23% of all included articles. Notably, Cattin, Philippe C (*n* = 23) from the University of Basel emerged as the most prolific author, followed closely by Zam, Azhar (*n* = 17) from the University of Basel/New York University. Among these top contributors, Sader, Robert achieved the highest H-index score of 11 while Gangi, Afshin from CHU Strasbourg garnered an impressive total citation count of 567. Additionally, Aoki, Akira from Tokyo Medical & Dental University (TMDU) attained the highest average number of citations at an impressive value of 74.43.

After setting the entry threshold at a minimum of 5 publications, only 31 out of 1,507 authors fulfilled this criterion. The network visualization map (Figure 4B) illustrates the co-authorship analysis among these selected authors using VOSviewer. If we exclude authors who have no connections with other authors, only 15 out of the initial group of 31 can be displayed. Among them, Cattin, Philippe C (56), Zam, Azhar (52), and Canbaz, Ferda (29) exhibit the highest TLS.

**Figure 6:**
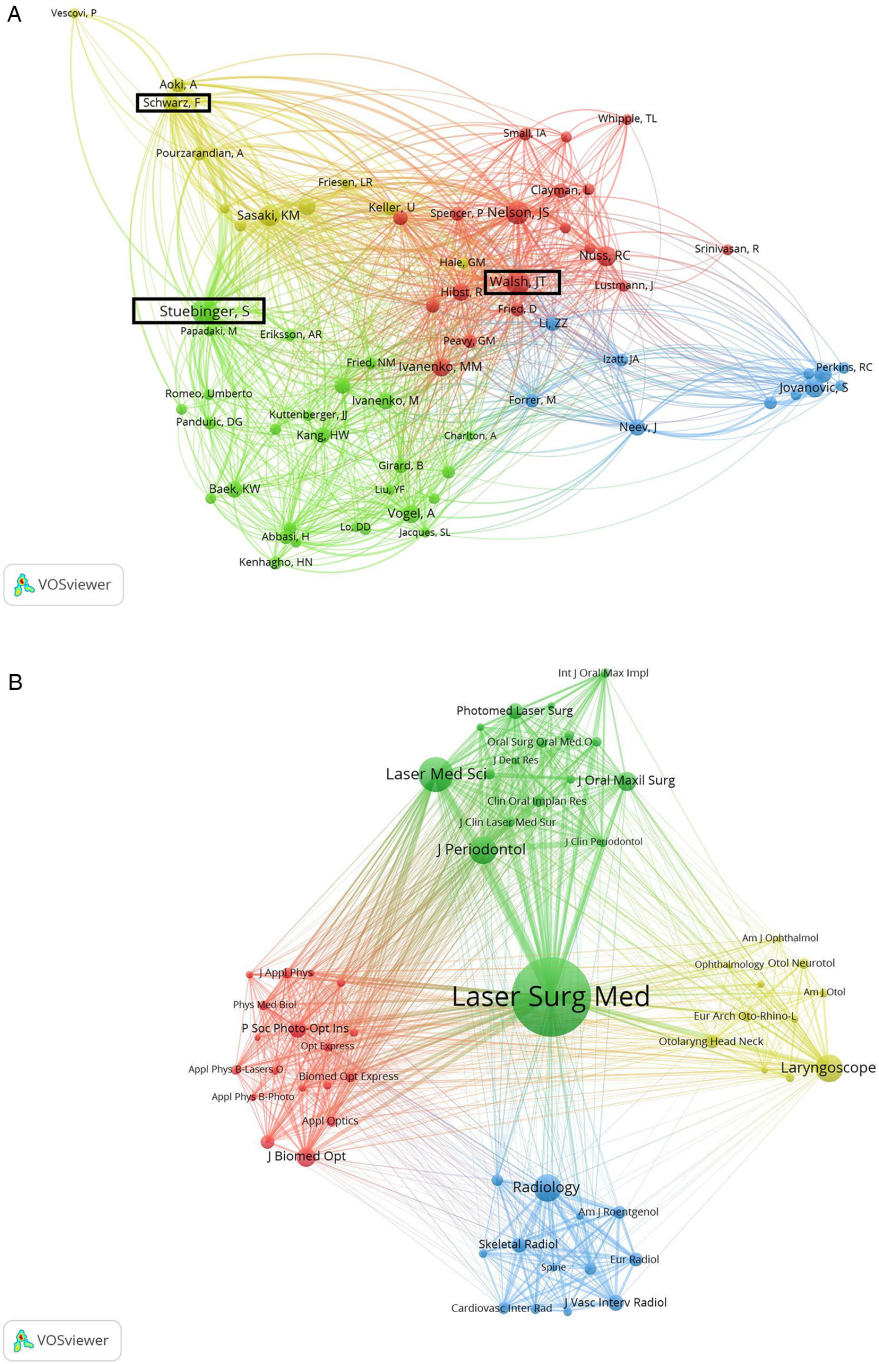
Co-citation analysis of global publications on laser bone ablation from 1979 to 2023. **(A)** Network visualization map of the top 75 authors identified in the laser bone ablation research. Authors bounded by black squares are authors with the highest TLS. **(B)** Network visualization map of the 59 journals involved in the laser bone ablation research. In the co-citation analysis map, the circle size of indicates the number of citations. The thickness of the lines represents the link strength between the items.

In addition, co-citation analysis is a method used to assess the relevance of items based on the frequency of being co-cited of an item (23). By setting the entry threshold at a minimum of 20 citations per author, 75 out of the total 5,942 authors met this criteria. The network visualization map of co-citation analysis for these 75 authors was generated using VOSviewer (Figure 6A), in which the circle size of indicates the number of citations and the thickness of the lines represents the link strength between the items. Additionally, in the co-citation analysis graph, the TLS represents the influence of each item. The top three authors with the highest TLS are Stuebinger, Stefan (1,383), Schwarz, Frank (1,220), and Walsh, Joseph T (1,192). Therefore, Stuebinger, Stefan can be considered as the most influential author in this field.

### 3.4 Analysis of institutions

A total of 511 institutions participated in the study on laser bone ablation. As depicted in Supplementary Table 3 and Figure 3C, the University of Basel made the most significant contribution by publishing 39 articles (10.18%), followed by Harvard University and the University of California System, which published 26 articles each (6.79%). Notably, Harvard University emerged as the institution with the TLS (1,100) and H-index (17). Furthermore, CHU STRASBOURG exhibited the highest average citation frequency of 54.45, closely followed by HARVARD MEDICAL SCHOOL with an average citation frequency of 45.40.

The network visualization map of the co-authorship analysis of the participating institutions was generated by VOSviewer, with an entry threshold set at 5 articles, and a total of 26 institutions were included (Figure 4C). Excluding those without links to other institutions, a total of 12 institutions are presented in the figure. The top three organizations with the highest TLS are: the University of Basel (37), Universitatsklinikum Basel (34), and the University of Zurich (21). Figure 4D represents an overlay visualization map showcasing the co-authorship analysis among institutions involved in this field, where circle colors indicate the average year of literature publication. Circles appearing bluer signify earlier document publications while circles appearing yellower represent later document publications (25). In recent years, Adv Osteot Tools AOT AG, Medical University of Vienna, New York University, and University of Basel have been more active, and there have been many collaborations between them (26–28).

### 3.5 Analysis of journals

Journals with a minimum of 10 published articles are presented in Supplementary Table 4, and the impact factor (IF) and quartile (Q) in the Category of each journal were obtained using Journal Citation Reports 2022. The journal that had the highest number of publications was *Lasers in Surgery and Medicine* (*n* = 50), which ranked first in terms of total times cited, average citations per item, and H-index. *Lasers in Medical Science* ranked second only to *Lasers in Surgery and Medicine* regarding the number of total publications, total times cited, and H-index.

In Figure 6B, the minimum number of citations of a journal was set at 40 and the co-citation analysis of 59 journals revealed that the three most influential journals in terms of TLS were *Lasers in Surgery and Medicine* (*n* = 26,792). Following closely were the *Journal of Periodontology* (*n* = 15,566) and *Lasers in Medical Science* (*n* = 13,651).

**Figure 7:**
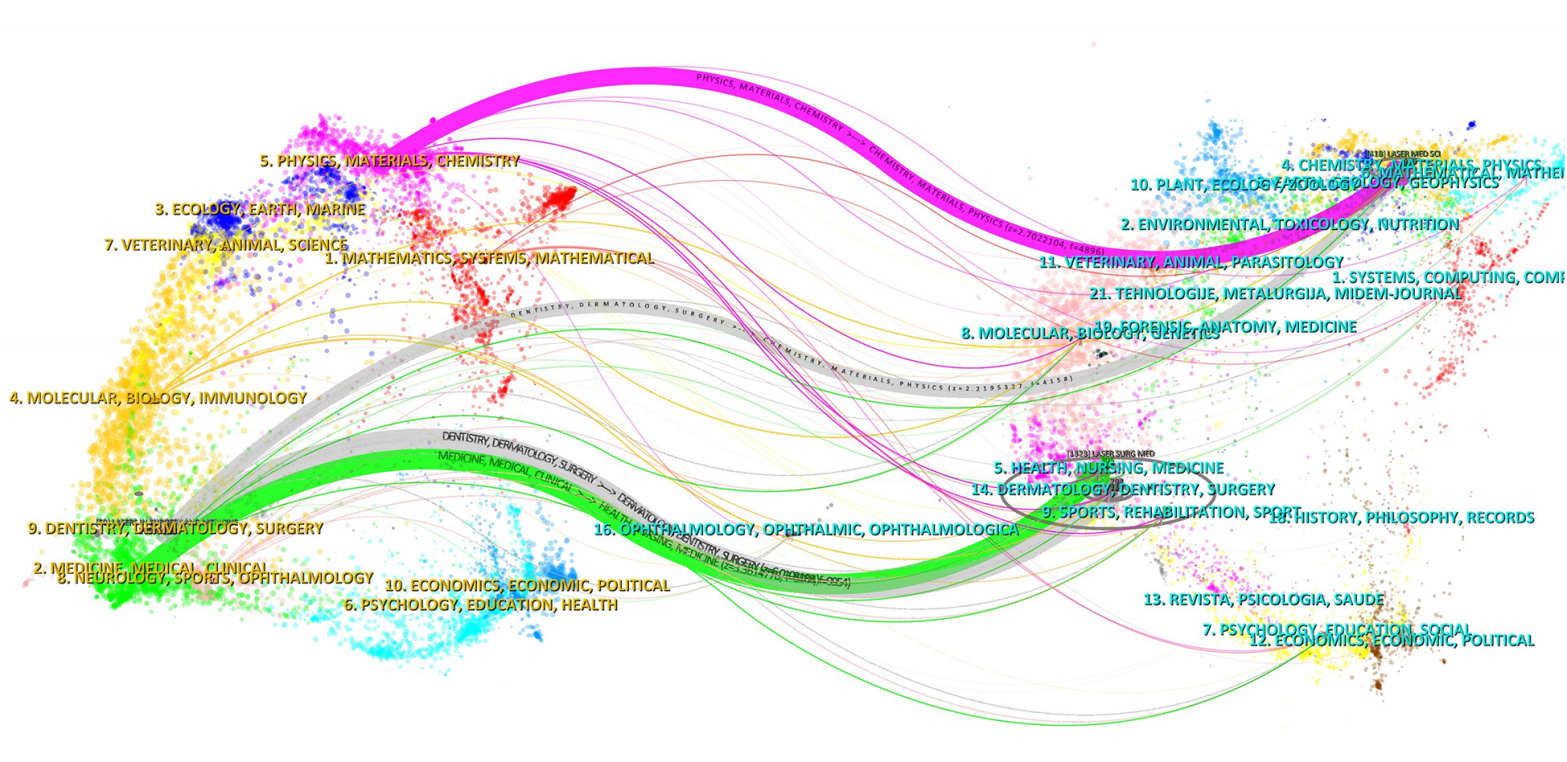
The dual-map overlay of journals stands for the topic distribution of academic journals. The citing journals are on the left, and the cited journals are on the right. The colored path represents the citation relationship.

The dual-map overlay of journals illustrates the degree of cross-fusion between the subject distributions of these academic journals (Figure 7) and the expansion and deepening between different research results. Citing journals and cited journals are positioned on the left and right sides, respectively, with the colored path representing the citation relationship. As can be seen from Figure 7, there are four primary pathways connecting cited journals and citing journals. Specifically, the strongest citation relationships were from Dentistry/Dermatology/Surgery journals to Dermatology/Dentistry/Surgery journals. The two fields are the same, which indicates that laser bone ablation still needs to be further cross-fused with other disciplines to generate more cross-disciplinary applications and theories.

### 3.6 Analysis of co-cited references and citation burst

**Figure 8:**
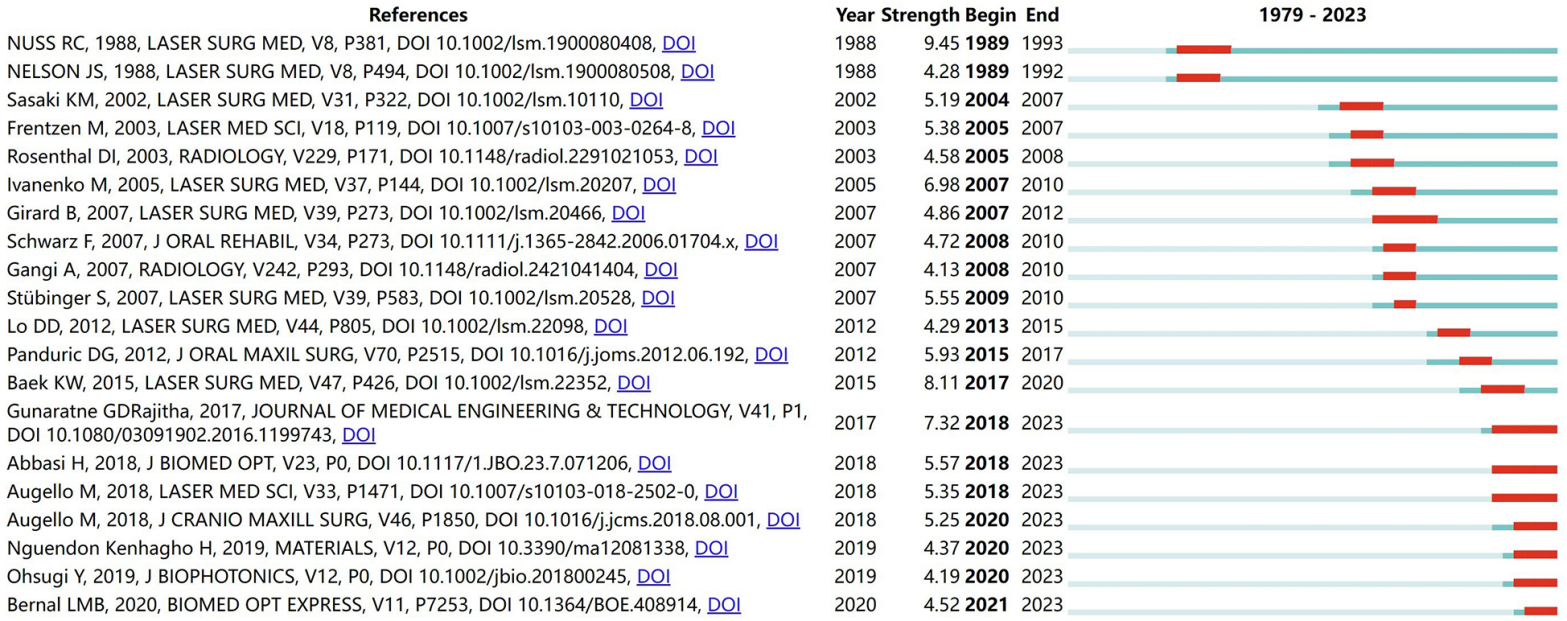
The top 20 references with the highest burst strength in the laser bone ablation research from 1979 to 2023. Years in light green mean that the reference has not yet appeared, years in dark green mean that the reference is less influential, and years in red mean that the reference is more influential.

The cited references underwent a comprehensive co-citation analysis. Out of the 7,967 references cited, 39 references were cited at least 20 times. Supplementary Table 5 reveals that the top 10 co-cited references had a minimum of 33 co-citations. References experiencing a citation burst are characterized by a significant increase in citation frequency over time (29, 30). The citation burst indicates that the corresponding research has rapidly garnered substantial attention from scholars and serves as an important indicator of research hotspots and frontiers within a specific field (31). Figure 8 presents the top 20 references with the highest burst strength. Years in light green signify that the reference has not yet appeared, while years in dark green represent that the reference has less influence, and years in red mean that the reference has greater influence (30). The earliest and most intense bursts (strength = 9.45) occurred in a study titled “Infrared Laser Bone Ablation” (32), published by NUSS, RC et al., in *Lasers in Surgery and Medicine* in 1988 with a burst duration spanning from 1989 to1993. Additionally, seven references still exhibited burstiness (6, 7, 33–37). These articles primarily focus on various aspects including physiological and histological effects of laser bone ablation; laser bone ablation under different external conditions and parameters; comparison between laser ablation and other osteotomy methods; reduction and detection of thermal damage during laser bone ablation; bone healing after laser ablation; deep bone ablation using lasers; robot or computer-assisted laser bone ablation. It is noteworthy that two out of these seven articles pertain to robot-assisted laser bone ablation topics while another two are centered around minimizing thermal damage.

### 3.7 Analysis of keywords

The analysis encompassed a total of 1,544 keywords, with 41 exhibiting a minimum frequency of at least 10 occurrences and 13 displaying a minimum frequency of at least 30 occurrences. Supplementary Table 6 presents the top ten most frequently observed keywords. “Ablation” emerged as the predominant keyword, being utilized in a total of 114 instances, followed by “Er:YAG laser” (*n* = 77) and “bone” (*n* = 76).

**Figure 9:**
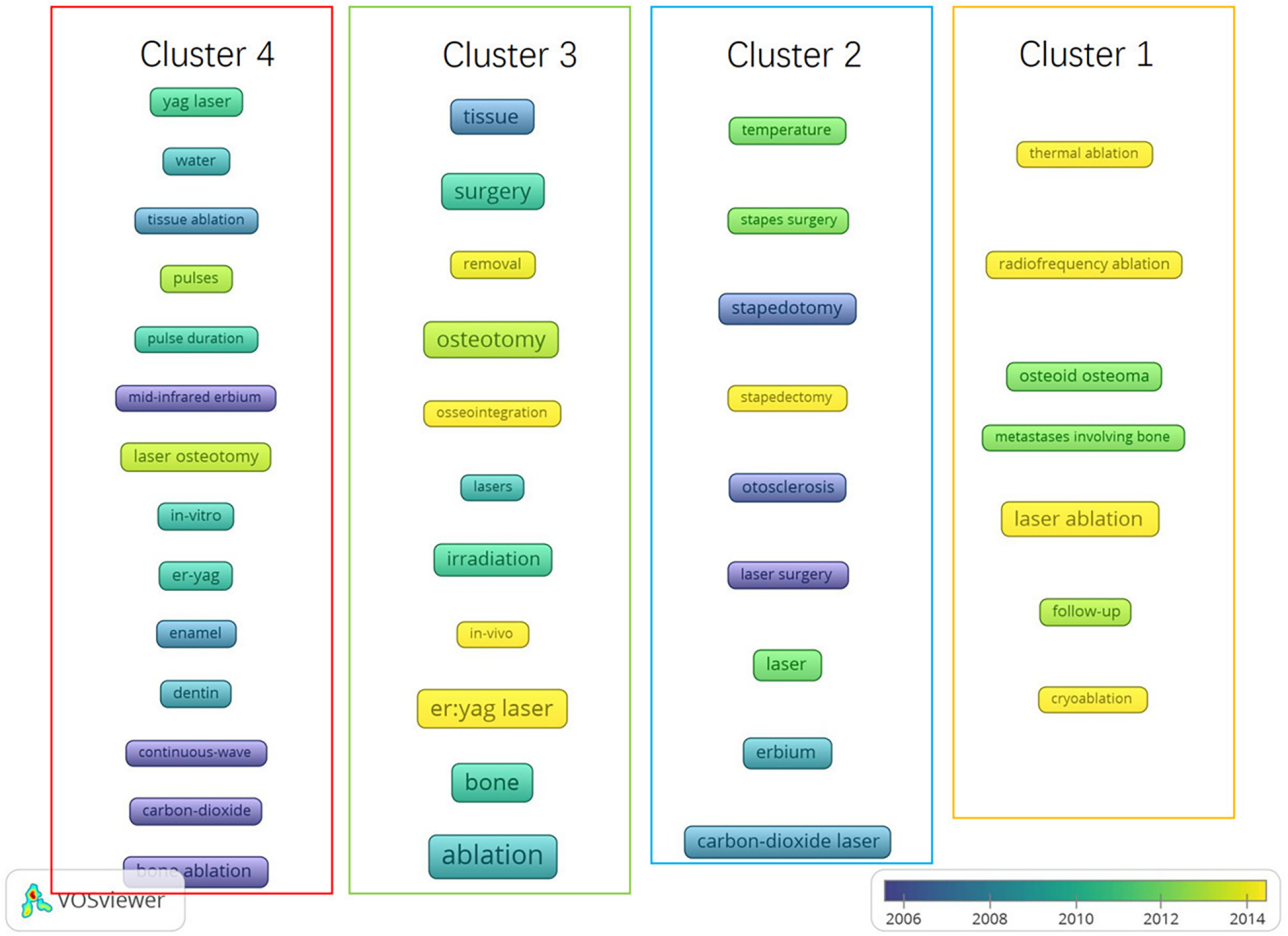
Co-occurrence analysis of global publications on laser bone ablation from 1979 to 2023. Overlay visualization map of the 41 keywords identified in the laser bone ablation research. Different clusters are framed by different colored squares, with the cluster name at the top.

The overlap visualization map of the co-occurrence analysis of the top 41 keywords (*n* ≥ 10) is presented in Figure 9. In this map, keywords with varying average appearance times are distinguished using different colors; Keywords with earlier average time of appearance are more blue, while keywords with later average time of appearance are more yellow. Co-occurrence analysis is a method used to identify high-frequency subject terms and research directions by quantifying the number of publications where different keywords co-occur (38, 39). In Figure 9, the four clusters are surrounded by boxes of different colors and labeled with names at the top. Cluster 1, Framed in yellow, encompasses high-frequency keywords such as “cryoablation,” “radiofrequency ablation,” “thermal ablation,” and “osteoid osteoma,” which are associated with comparison between laser ablation and other ablation methods of bone tumors. Cluster 2, Framed in blue, includes keywords like “stapedectomy”, “stapedotomy”, and “stapes surgery” that pertain to the application of lasers in stapes surgery. Cluster 3 Framed in green, consists of frequently occurring words such as “osseointegration,” “removal,” and “surgery” that relate to various applications of laser bone ablation. Lastly, cluster 4 is Framed in red and comprises high-frequency words like “pulse duration”, “continuous wave”, and “pulses” which are linked to different parameters utilized for laser bone ablation purposes.

**Figure 10:**
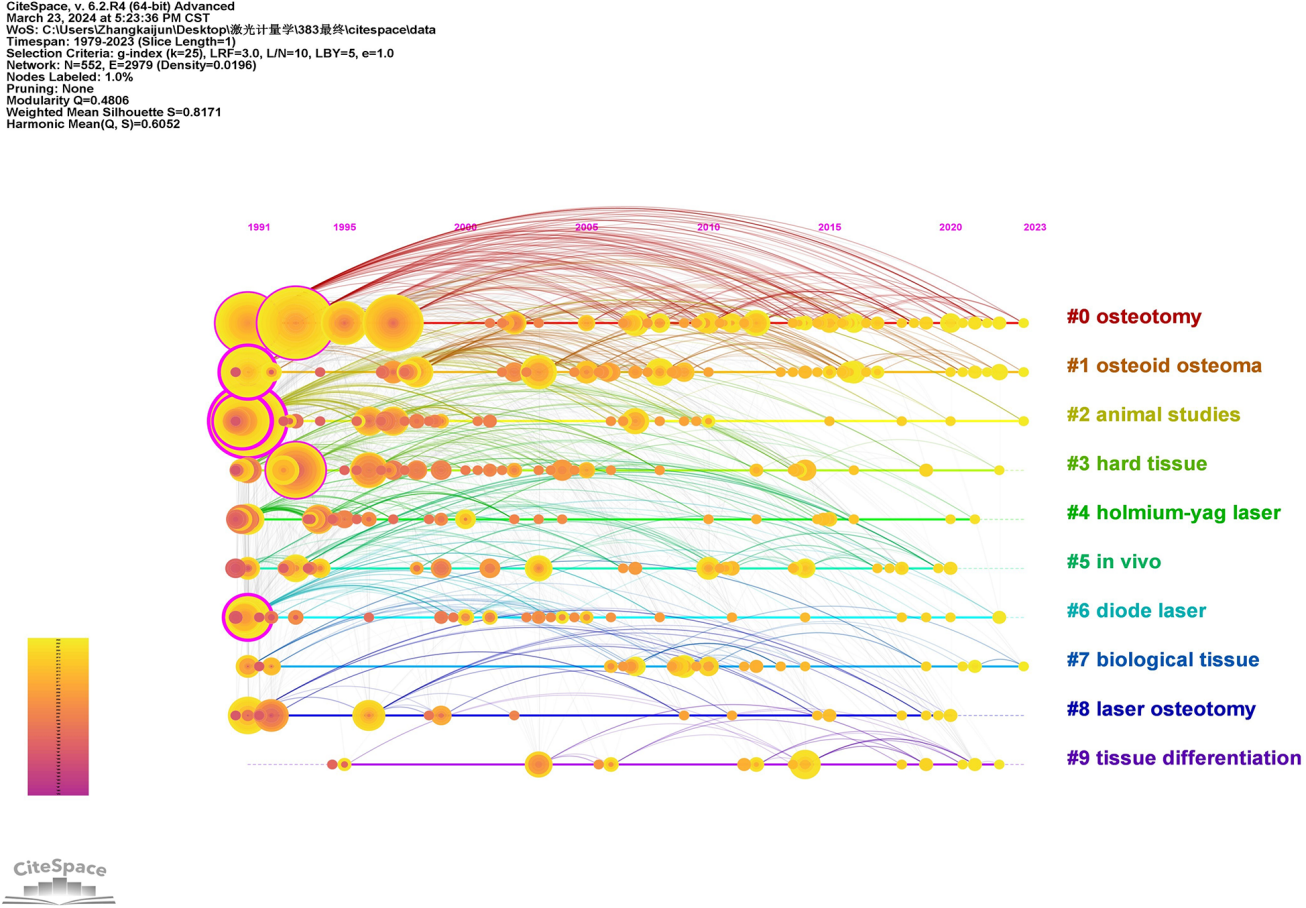
The timeline view of keywords related to the laser bone ablation research. The labels showing the time are arranged horizontally at the top, with the time range 1991–2023. The horizontal lines represent individual clusters, with labels denoting the name of each cluster at their respective ends. The size of the cluster is inversely proportional to its number, where #0 and #9 denote the largest and smallest clusters respectively. Cluster labels were extracted by LLR. Circles appear in the year of the first co-occurrence of the keyword. The circle's size reflects co-occurrence frequency, while the link represents co-occurrence of the relationship.

We utilized CiteSpace to generate a timeline visualization of the keywords (Figure 10) in order to further investigate the temporal evolution characteristics of distinct clusters (40). Figure 10 illustrates a total of ten clusters, with different colors representing each cluster. The size of the cluster is inversely proportional to its number, where #0 and #9 denote the largest and smallest clusters respectively. The names of the top five keyword clusters identified were “osteotomy,” “osteoid osteoma,” “animal studies,” “hard tissue,” and “holmium-yag laser.” This figure clearly depicts the time points at which these ten keyword clusters emerged. Labels denoting the name of each cluster are presented at the end of their respective timelines. Time labels are arranged horizontally at the top, covering a time range from 1991 to 2023. Circles appear in years when keywords first co-occurred, with circle size reflecting co-occurrence frequency and links indicating co-occurrence relationships.

## 4 Discussion

In this study, a total of 383 articles were identified from the WOS database spanning the years 1979 to 2023. These articles encompassed contributions from 1,508 authors, affiliated with 511 institutions across 44 countries/regions, and were published in 154 journals. Additionally, we analyzed a corpus of 7,967 cited articles sourced from 2,510 journals. Employing bibliometric analysis methodology, this paper provides comprehensive insights into laser bone ablation from various perspectives. The findings presented hold significant implications for guiding future practices and advancing the development of this field.

### 4.1 Country

Research in this field is primarily distributed across North America, Central Europe, Western Europe, and East Asia. The United States leads the way with the earliest publication, the highest number of total publications, and extensive collaboration with other countries in this field. It also holds the highest H-index. Its most significant contributions to this field in terms of quantity, quality, and power may be closely tied to its advanced level of scientific and technological development, substantial economic influence, and extensive investment in healthcare. Figure 2A illustrates a rapid increase in international publications since 2018. Over the past 5 years, these publications account for 25.33% of the total publications—a contribution largely influenced by Switzerland's involvement (Figure 2B). The strong precision manufacturing industry in Switzerland may account for its consecutive first ranking worldwide in terms of annual publications from 2018 to 2022. Among the top ten countries in terms of total publications, the People's Republic of China is the sole developing country (Supplementary Table 1), potentially attributed to the substantial implementation costs associated with laser surgery due to equipment and maintenance expenses (41). The network visualization map of co-authorship analysis reveals eight clusters (Figure 4A). In the yellow cluster, the link strength of Austria, Germany, and Switzerland is strong, indicating a high level of cooperation, possibly because these three countries are adjacent in geographical position. The People's Republic of China, Japan, and Canada form part of the green cluster. These three countries are all among the top globally in terms of total publications. Notably, the United States, Switzerland, and Germany—which hold leading positions worldwide in both total publications and H-index—exhibit strong interconnections with each other. It may be that the cooperation between countries promotes the output and quality of each other's scientific research.

### 4.2 Author and institution

The analysis of influential authors and institutions helps scholars to understand domestic and foreign partnerships and find potential partners (42). According to Supplementary Table 2, Cattin, Philippe C, Zam, Azhar, and Sader, Robert are the most prolific contributors in this field. Additionally, Cattin, Philippe C, and Sader, Robert possess the highest H-index. Cattin, Philippe C emerges as the most cooperative author. Among the top seven authors with significant contributions, five are affiliated with the University of Basel in Switzerland. Stuebinger, Stefan, the most influential author in this field, also from the University of Basel, conducted a study titled “Comparison of Er:YAG Laser and Piezoelectric Osteotomy in 2010: An Animal Study in Sheep”. The study demonstrated that Er:YAG laser can effectively be utilized to perform an osteotomy up to a depth of 22 mm in the sheep tibia without any thermal damage, challenging the prevailing belief of adverse effects of laser osteotomy due to thermal damages (43).

In the Network visualization map of co-authorship analysis (Figure 4B), the largest cluster is represented by the red cluster, which comprises only seven authors. Additionally, the green cluster includes Sader, Robert, Stuebinger, Stefan and Zeilhofer, Hans-Florian, all of whom are the top six authors in terms of total publications. Moreover, Sader, Robert, and Stuebinger, Stefan are the authors with the highest H-index and the most influential respectively. It is possible that the cooperation between authors and the improvement of the quantity, quality, and influence of the authors can promote each other. However, no link exists between the green cluster and the blue cluster. Consequently, it is imperative for authors to reinforce cooperation within this domain.

Among the top five institutions in terms of total publications, four are affiliated with the United States. Additionally, three out of the top four institutions in terms of H-index also belong to the United States (Supplementary Table 3). The map of co-authorship analysis was partitioned into five clusters, with the red and green clusters being the largest ones (Figure 4C). Notably, the University of Basel emerged as the most cooperative institution, closely followed by Universitatsklinikum Basel. The publications of institutions in the red cluster were published later than those of other clusters, as evidenced by the comparison between the network visualization map and overlay visualization view (Figure 4D). This delay could potentially be attributed to collaborative efforts among institutions fostering innovation promotion.

### 4.3 Journals and references

In terms of the quantity of published articles, only six journals have published a minimum of 10 articles, with the top four being exclusively laser medicine or biomedical optics journals (Supplementary Table 4). Among these six journals, the highest JCR partitioning is Q2, and the journal with the most significant impact factor is the *Journal of Biomedical Optics* (IF = 3.5), indicating that research in this field is limited and lacks recognition from high-impact medical journals.

The network visualization map of Co-citation analysis reveals that *Lasers in Surgery and Medicine* is the most influential journal (Figure 6B), exhibiting the largest number of publications and the highest H-index. It holds JCR partitions Q2 and Q3 for surgery and dermatology respectively, publishing basic and clinical research on laser applications in various surgical and medical specialties. The *Journal of Periodontology* ranks high in terms of influence, placing second, likely due to the exceptional performance of Er:YAG laser in periodontal hard tissue surgery. This can also be attributed to the fact that this laser is regarded as one of the most promising lasers in periodontal treatment (44). The JCR partition of the *Journal of Periodontology* in Dentistry, Oral Surgery & Medicine was Q1 (2022), and the impact factor was 4.3 (2022), so it has a high professional recognition in the field of stomatology. Notably, *Lasers in Medical Science* ranks third in impact while securing second place both in terms of total publications and H-index. This journal focuses on technical, experimental, and clinical aspects related to laser utilization in medicine.

Co-citation analysis of publications can identify research hotspots in a specific field. Among the top 10 most frequently cited articles, seven were from *Lasers in Surgery and Medicine* (Supplementary Table 5). Eight of the ten studies studied the use of Er:YAG laser in bone ablation, including measurements of the ablation efficiency of Er:YAG laser with different parameters on bone tissue (45), thermal and acoustic effects of Er:YAG laser ablation of bone (14), thermal damage of Er:YAG laser ablation of bone (46), morphological characteristics and chemical composition of bone surface after Er:YAG laser ablation (32, 47, 48), and the bone healing of Er:YAG laser osteotomy compared with that of drilling osteotomy or mechanical saw osteotomy (49, 50). One paper studies the bone ablation mechanism of carbon dioxide laser with different pulse durations and wavelengths (51), and another paper compares the effect of continuous-wave and rapid superpulsed carbon dioxide laser osteotomy on bone healing (52).

### 4.4 Keywords and research trend

Through the co-occurrence analysis of keywords, it can be seen that the current research on laser bone ablation is mainly focused on the study of laser bone ablation parameters, laser bone tumor ablation, laser stapes surgery, and various applications of laser bone ablation.

Firstly, wavelength, pulse duration, and power are crucial parameters of lasers that significantly impact laser bone ablation. Pulse duration affects the mechanism of ablation. Forrer et al. (51) employed a CO_2_ laser for ablating pig ribs and investigated the ablation mechanism through light and electron microscopy analysis at short pulse durations of 0.9 and 1.8 μs, as well as long pulse duration of 250 μs. Their findings revealed that under short pulse durations, ablation primarily relied on the explosive evaporation of water, whereas under long pulse duration, the driving force of the ablation process was attributed to the absorption of CO_2_ radiation by the carbonized layer. Majdani et al. (53) also used a CO_2_ laser to ablate the cochlea of a corpse and obtained an empirical formula for ablation Depth: Depth (in mm) = 0.84 (duration of laser in msec) + 0.40 (power in W). Different from the study by Majdani et al., Peavy et al. (54) proposed that laser bone ablation is dependent on wavelength. The bovine cortical bone was subjected to ablation using a free electron laser operating at a specific wavelength range of 2.9–9.2 μm, revealing that the most profound ablation pits were observed within the wavelength range of 6.1–6.45 μm. In the infrared spectrum, water exhibited an absorption peak at 6.1 μm, while protein displayed absorption peaks at both 6.06 and 6.45 μm wavelengths respectively. At a wavelength of 3.0 μm, where only water demonstrated an absorption peak, the cutting depth was found to be smaller; however, ablative surfaces remained clean without thermal damage at this specific wavelength (3 μm). Youn et al. (55) employed four different wavelengths generated by a free electron laser for bovine cortical bone ablations: 2.9, 6.1, 6.45, and 2.79 μm. Their results indicated that under equal laser fluence, the highest efficiency in terms of ablation and lowest thermal damage occurred at *λ* = 6.1 μm, while the lowest efficiency in terms of ablation and highest thermal damage occurred at *λ* = 2.79 μm. Peavy and Youn both identified the same wavelength as producing the deepest part of the ablation pit; specifically when it was set at 3.0 μm, less thermal damage was observed. In recent years, Er:YAG laser with a wavelength of 2.94 μm, which is similar to 3 μm, has also been widely used because of its advantages of less thermal damage.

In laser bone tumor ablation, the principle involves inserting an optical fiber into the lesions of the bone tumor and transmitting infrared energy to the tumor through the exposed tip of the optical fiber. The bare tip of the optical fiber serves as a heat source, rapidly increasing temperature and causing denaturation and coagulated necrosis of the protein in the bone tumor, primarily including Laser photocoagulation or laser interstitial thermotherapy (56, 57). This ablation method offers advantages such as high precision and minimal impact on adjacent tissues (58). The laser ablation instrument is compatible with MR imaging, allowing for easy utilization of MR guidance (59). Furthermore, Shanmugasundaram et al. (60) conducted a meta-analysis evaluating percutaneous ablation methods for osteoid osteoma and reported that laser ablation had significantly shorter operation times compared to radiofrequency ablation, cryoablation, and microwave ablation (56). However, this method has limitations including a small range of ablation and limited penetration depth (58). Real-time monitoring within tumors is not possible with bare fiber tips (59). Currently, laser ablation is primarily used for treating osteoid osteoma and osteoblastoma; it is also one of the preferred treatment modalities for these two tumors. Laser ablation demonstrates low complication rates and high efficacy in treating Osteoid Osteomas. Gangi et al. (61) treated 114 cases using laser ablation for OOs which proved to be effective in 112 patients whose pain relieved within 1 week and the 114 patients did not experience any significant complications, such as pathological fractures, neurovascular or adjacent tissue injuries, or infections. Additionally, previous studies have shown that (62) the ablation area size of bone tumors depends on the laser wavelength and power used, the thermal and optical properties of the target tissue, the duration of energy deposition, and the laser fiber diameter, independent of the laser pulse width. A 980 nm diode laser and 1,064 nm neodymium-doped yttrium aluminum garnet (Nd: YAG) ensure optimal absorption and penetration rates (63). The duration of laser ablation is usually 200–600 s, depending on the size of the target area. This formula is commonly used to determine the amount of energy required to achieve complete ablation: tumor size (mm) × 100 J + 200 J.

In terms of laser stapes surgery, stapes surgery is considered the gold standard for otosclerosis (64). Lasers in stapes surgery are now used to divide the stapedius tendon, divide the anterior and posterior crus, and perforate the footplate. Currently, various lasers including Argon laser, KTP laser, CO_2_ laser, Er:YAG laser, Diode laser, thulium laser, and Ho: YAG laser can be used in stapes surgery. Each of these lasers has its own advantages and disadvantages. The ideal laser for stapes surgery should not penetrate into the perilymph to avoid increasing its temperature. It should also be able to transmit through optical fibers for easy operation and efficiently absorb water for high bone ablation efficiency (65). The Er:YAG laser is the first choice of laser for stapedotomy, due to its limited optical penetration depth in water, the parameters of which were 0.25 ms pulse width in the stape surgery. The power of the Stapes crura and Stapes footplate at the time of operation was 60MJ and 30–60MJ (65). Consequently, it enables precise ablation of bone tissues with high water content and minimizes thermal damage to surrounding tissues, making it suitable for middle ear surgery involving complex structures. Additionally, studies have confirmed that the use of Er:YAG laser does not result in a rapid increase in the temperature of inner ear lymph fluid (66). However, unlike the CO_2_, KTP, Ho: YAG, argon laser, etc. used in middle ear surgery, the ablation of Er:YAG laser is achieved by microexplosion rather than thermal effect (67). Häusler et al. (66) used Er:YAG laser to perform stapedotomy on 3 patients, the bone conduction threshold decreased by 75 dB in medium and high frequency 2 h after surgery, and this threshold shift returned to close to the preoperative value within 6 h, because the explosive ablation of tissue by Er:YAG laser pulse would produce pressure waves and cause acoustic damage. Nagel et al., who first reported the clinical application of the Er:YAG laser in otologic surgery, suggested that the Er:YAG laser has the potential to become a useful tool in middle ear surgery. Combined with the animal experiment of Ruedi et al. (68), they believed that there was no hearing loss at an Er:YAG laser dose below 25,000 mJ, sufficient to remove bone tissue equivalent to the mass and size of the malleus or incus, and this dose was also defined as the maximum acceptable dose for ear surgery (67). McCaughey et al. (69) used femtosecond laser ablation of porcine otic capsule bone to simulate stapes laser surgery and found that no signs of thermal damage or carbonization were observed in the ablation pits of the femtosecond laser, the bottom and wall of the pits were smoother and clearer than those of the Er:YAG and any photoacoustic stress or sound of the femtosecond laser was smaller, even smaller than the range of the detector and could not be recorded. As a result, femtosecond lasers are showing a trend to replace Er:YAG lasers.

Laser bone ablation has a wide range of applications, including osteo-oncology and otologic surgery. Additionally, it is worth noting the application of laser bone ablation in maxillofacial surgery, which has not only demonstrated favorable clinical outcomes but also successfully integrated cutting-edge technology. Stuebinger et al. (70) utilized Er:YAG laser ablation to treat 8 patients with bisphosphonate medication-related osteonecrosis of the jaw. The surgical procedure and postoperative wound healing proceeded without any complications, and a complete recovery of soft tissue was achieved within 4 weeks. Ureel et al. (27) employed the cold ablation robot-guided laser osteotome (CARLO(R)) for the first time on a human body to perform the linear part of the Le Fort I osteotomy under direct visual control, also leading to good wound healing without complications post-surgery. Furthermore, laser bone ablation shows potential clinical advantages in assisting implant surgery. Kesler et al. (71) used Er:YAG laser and drill respectively for osteotomy and titanium alloy implant placement on sheep tibias. It was found that the Er:YAG laser can obtain good osseointegration results and bone healing in implant site preparation. Moreover, the percentage of bone-implant contact (BIC) is higher significantly compared to that achieved with drill.

The overlay visualization map enables the prediction of emerging research topics and facilitates monitoring progress in the field. Based on Figure 9, the yellow items represent the research directions that emerged after 2014. It contains “cryoablation” and “thermal ablation” related to bone tumors. It is possible that articles related to laser ablation of bone tumors tend to compare various bone tumor ablation methods. Percutaneous minimally invasive image-guided interventions have made substantial progress in the treatment of bone tumors, including radiofrequency ablation, cryoablation, microwave ablation, laser photocoagulation, etc., and the safety, efficacy, and durability of these interventions have been proven (62), so these techniques will be introduced or compared together. Additionally, “stapedectomy”, “osseointegration” and “*in vivo*” are all shown as yellow items. “Stapedectomy” is associated with laser stapedotomy, and “osseointegration” pertains to the laser preparation of implants for dental implant surgery. Both procedures necessitate precise cutting techniques. Lasers enable the cutting of tissue with a highly focused area of action, minimizing damage to adjacent tissues (65, 72), aligning with the contemporary trend towards precision in medical practice. Additionally, “*in vivo*” pertains to studies conducted within living animals. Perhaps due to the lack of clinical studies on laser bone ablation, it is imperative to validate this technique through animal experiments prior to its application in clinical settings (73).

The timeline view also includes time parameters, facilitating our comprehension of the research hotspots in different periods (42). As depicted in Figure 10, the current research regarding laser bone ablation focuses on “osteotomy (#0)”, “osteoid osteoma (#1)”, “animal studies” (#2), and “biological tissue (#7)”. This indicates that the present hotspots revolve around laser osteotomy (74), laser ablation of bone tumors (62), animal experimentation (75), as well as the interaction with biological tissues during laser bone ablation (76). Based on the analysis results of the above overlay visualization map, laser osteotomy, laser bone tumor ablation, animal experiments and the interaction with biological tissues during laser bone ablation may be the hot spots and directions in this field in the future.

Regarding laser ablation of bone tumors, future studies will focus on the development of percutaneous minimally invasive image-guided interventions (62). Regarding laser osteotomy, future research trends will focus on precise cutting, which is reflected in the control of cutting depth and the differentiation of tissue components. Seppi et al. (77) found that during ablation, each pulse emits sound waves, which can be captured by an air-coupled transducer, and the data is used to predict the depth of cutting during ablation. Kenhagho et al. (78) used acoustic shock waves generated by lasers during ablation to classify sciatic nerve tissue with other tissue types (hard bone, cartilage, fat, muscle, and skin extracted from the proximal and distal ends of the pig femur). In addition, Bayhaqi et al. (79) proposed that optical coherence tomography (OCT) images identify tissue types and provide feedback for laser ablation, thereby avoiding critical tissues such as bone marrow and nerves. With regard to the interaction between laser and biological tissues and animal experiments, attention should continue to be paid to reducing complications such as thermal damage in the future. Ji et al. (80) propose a novel laser fast and safe drilling strategy with dynamic focusing and diffused droplet cooling. Within 30 s, smooth and clean through-holes with a diameter of 3 mm and a depth of 4 mm were successfully drilled on the tibia of sheep *in vitro*, and the bone temperature was kept below 47℃. Dragana et al. (81) conducted an *in vivo* experimental animal study using rats and found that the use of Er:YAG laser osteotomy in contact mode reduced the potential overheating of bone tissue compared with the use of non-contact Er:YAG laser osteotomy.

### 4.5 Limitations

The study employed bibliometric analysis and publication visualization methods to objectively depict the research trend and status of laser bone ablation-related publications. Nevertheless, there are certain limitations in this investigation. Firstly, the bibliometric analysis was conducted solely based on the WOSCC database. Web of Scienceis a large international authoritative database that can cover basic data (82). Although we tried to integrate other databases, bibliometrics analysis software has high specifications and standards for data, and many databases cannot effectively export bibliometrics data that can be comprehensively analyzed. To ensure the quality and integrity of the collected data, only the Web of Science database was selected in this study, excluding other databases. Secondly, only English articles and reviews were included in this study. English is the most mainstream academic publication language, and the quality of publications written in English is more guaranteed than publications in other languages. Lastly, VOSviewer and CiteSpace lack the capability to analyze the full-text content of publications, which may result in potential information omission, but According to Pan et al. (83), despite certain biases in comprehensiveness, VOSviewer and CiteSpace are still frequently and widely used, indicating the high value and status of this two software in practical applications. After all, keywords cover most of the key information, which also makes the analysis more focused on the key information.

## 5 Conclusion

The field of laser bone ablation has witnessed a consistent upward trend in the annual number of articles. Although the number of articles published before 1996 was at a low level, there has been a significant increase in the number of articles published from 2019 to 2021, with 2021 recording the highest publication count. The United States is the country with the earliest publication, the largest number of publications, and the closest cooperation with other countries in this field. It has contributed the most to this field in terms of quantity and quality. Among influential journals, *Lasers in Surgery and Medicine* holds prominence with its vast publication volume. Research on laser bone ablation primarily focuses on the parameters of laser bone ablation, laser bone tumor ablation, laser stapes surgery, and diverse applications related to laser bone ablation. The laser osteotomy, laser ablation of bone tumors, animal experiments, and the interaction with biological tissues during laser bone ablation are expected to be the focal areas and future directions in this field.
