# Supplementary material for: Global trends of laser bone ablation: bibliometric analysis of publications from 1979 to 2023
Source: Front Surg. 2025 Mar 11;12:1461319. doi: 10.3389/fsurg.2025.1461319 (PMC11933057; doi:10.3389/fsurg.2025.1461319)
Supplement: Supplementary file 1 [file Datasheet1.zip › Table 1.DOCX]

| **Rank** | **Country/Region** | **Articles** | **Count（%）** |
| --- | --- | --- | --- |
| 1 | USA | 124 | 32.3 |
| 2 | Germany | 74 | 19.3 |
| 3 | Switzerland | 53 | 13.8 |
| 4 | France | 25 | 6.5 |
| 4 | United Kingdom | 25 | 6.5 |
| 4 | Peoples R China | 25 | 6.5 |
| 7 | Canada | 21 | 5.5 |
| 8 | Italy | 21 | 5.5 |
| 9 | Japan | 17 | 4.4 |
| 10 | Austria | 16 | 4.2 |

Supplementary Table 1 | The top 10 countries/regions that contributed to the laser bone ablation research.

| **Author** | **Institution** | **Country** | **Total publications*10** | **Total times cited** | **Average citations per item*10** | **H-index*10** | **Self-citation times*10** |
| --- | --- | --- | --- | --- | --- | --- | --- |
| Cattin, Philippe C | University of Basel | Switzerland | 230 | 296 | 128.7 | 110 | 650 |
| Zam, Azhar | University of Basel/New York University | Switzerland/USA | 170 | 168 | 98.8 | 80 | 380 |
| Sader, Robert | Goethe University Frankfurt | Germany | 120 | 357 | 297.5 | 110 | 250 |
| Zeilhofer, Hans-Florian | University of Basel | Switzerland | 110 | 332 | 301.8 | 100 | 250 |
| Gangi, Afshin | CHU Strasbourg | France | 100 | 567 | 567 | 100 | 170 |
| Stuebinger, Stefan | University of Basel | Switzerland | 100 | 284 | 284 | 90 | 170 |
| Canbaz, ferda | University of Basel | Switzerland | 90 | 62 | 68.9 | 50 | 50 |
| Hering, P. | Heinrich Heine University Dusseldorf | Germany | 80 | 288 | 360 | 70 | 170 |
| Guzman, Raphael | University of Basel | Switzerland | 80 | 93 | 116.3 | 50 | 150 |
| Rauter, Georg | University of Basel | Switzerland | 70 | 78 | 111.4 | 50 | 40 |
| Wong, Brian J-F | University of California Irvine | USA | 70 | 75 | 107.1 | 60 | 80 |
| Aoki, Akira | Tokyo Medical & Dental University | Japan | 70 | 521 | 744.3 | 70 | 150 |

Supplementary Table 2 | The top 12 authors that contributed to the laser bone ablation research.

、

Supplementary Table 3 | The top 10 institutions involved in this research.

| **Rank** | **Institution** | **Country** | **Total publications** | **Count (%)** | **Total times cited** | **Average citations per item** | **H-index** | **Self-citation times** |
| --- | --- | --- | --- | --- | --- | --- | --- | --- |
| 1 | University of Basel | Switzerland | 39 | 10.18 | 651 | 16.69 | 16 | 137 |
| 2 | Harvard University | USA | 26 | 6.79 | 1110 | 42.69 | 17 | 24 |
| 2 | University of California System | USA | 26 | 6.79 | 918 | 35.31 | 16 | 60 |
| 4 | University of California Irvine | USA | 19 | 4.96 | 691 | 36.37 | 13 | 42 |
| 5 | Harvard Medical School | USA | 15 | 3.92 | 681 | 45.40 | 11 | 10 |
| 6 | Goethe University Frankfurt | Germany | 14 | 3.66 | 366 | 26.14 | 11 | 21 |
| 7 | Massachusetts General Hospital | USA | 13 | 3.39 | 435 | 33.46 | 11 | 9 |
| 7 | University of Zurich | Switzerland | 13 | 3.39 | 281 | 21.62 | 10 | 13 |
| 9 | CHU Strasbourg | France | 11 | 2.87 | 599 | 54.45 | 10 | 19 |
| 10 | Heinrich Heine University Dusseldorf | Germany | 10 | 2.61 | 316 | 31.60 | 9 | 14 |

| Rank | Source | Total publications | Count（%） | IF（2022） | IF  （5 year） | Quartile in Category  （2022） | Total times cited | Average citations per item | H-index |
| --- | --- | --- | --- | --- | --- | --- | --- | --- | --- |
| 1 | *Lasers in Surgery and Medicine* | 50 | 13.05 | 2.4 | 2.7 | Q2/Q3 | 1997 | 39.94 | 27 |
| 2 | *Lasers in Medical Science* | 34 | 8.88 | 2.1 | 2.5 | Q2/Q4 | 602 | 17.71 | 14 |
| 3 | *Journal of Biomedical Optics* | 14 | 3.66 | 3.5 | 3.2 | Q2 | 269 | 19.21 | 12 |
| 4 | *Biomedical Optics Express* | 13 | 3.39 | 3.4 | 3.7 | Q2 | 158 | 12.15 | 8 |
| 5 | *Laryngoscope* | 12 | 3.13 | 2.6 | 2.7 | Q2/Q3 | 471 | 39.25 | 11 |
| 6 | *Photomedicine and Laser Surgery* | 11 | 2.87 | 2.796 | 2.964 | Q2 | 199 | 18.09 | 5 |

Supplementary Table 4 | Journals with at least 10 publications in the laser bone ablation research.

Supplementary Table 5 | The top 10 co-cited references related to the laser bone ablation research.

| **Title** | **First author** | **Journal** | **Year** | **Citations** |
| --- | --- | --- | --- | --- |
| Infrared-laser bone ablation | Nuss RC | *Lasers in Surgery and Medicine* | 1988 | 71 |
| Mid-infrared erbium - yag laser ablation of bone - the effect of laser osteotomy on bone healing | Nelson JS | *Lasers in Surgery and Medicine* | 1989 | 53 |
| Er yag laser ablation of tissue - measurement of ablation rates | Walsh JT | *Lasers in Surgery and Medicine* | 1989 | 44 |
| Use of the Er:YAG laser for improved plating in maxillofacial surgery: Comparison of bone healing in laser and drill osteotomies | Lewandrowski KU | Lasers In Surgery And Medicine | 1996 | 43 |
| Ultrastructural analysis of bone tissue irradiated by Er:YAG laser | Sasaki KM | *Lasers in Surgery and Medicine* | 2002 | 37 |
| Bone ablation with er-yag and co-2 laser - study of thermal and acoustic effects | Li ZZ | *Lasers in Surgery and Medicine* | 1992 | 35 |
| Healing of continuous-wave and rapid superpulsed, carbon-dioxide, laser-induced bone defects | Clayman L | *Journal of Oral Surgery* | 1978 | 34 |
| Bone-Ablation Mechanism Using CO2 Lasers of Different Pulse Duration and Wavelength | Forrer M | *Applied Physics B-Photophysics and Laser Chemistry* | 1993 | 34 |
|  |  |  |  |  |
| Ablation of Bone and Methacrylate by a Prototype Mid-Infrared Erbium:YAG Laser | Nelson JS | *Lasers in Surgery and Medicine* | 1988 | 33 |
| Scanning Electron Microscopy and Fourier Transformed Infrared SpectroscopyAnalysis of Bone Removal Using Er:YAG and CO2 Lasers | Sasaki KM | *Journal of Periodontology* | 2002 | 33 |

Supplementary Table 6 Top 10 co-occurrence keywords of the laser bone ablation research

| **Rank** | **Keywords** | **Occurrences** |
| --- | --- | --- |
| 1 | ablation | 114 |
| 2 | er:yag laser | 77 |
| 3 | bone | 76 |
| 4 | osteotomy | 70 |
| 4 | surgery | 70 |
| 6 | tissue | 55 |
| 7 | laser ablation | 53 |
| 8 | carbon-dioxide laser | 45 |
| 9 | irradiation | 41 |
| 10 | erbium | 39 |
