# Supplementary figures and images for: Global trends of laser bone ablation: bibliometric analysis of publications from 1979 to 2023
Source: Front Surg. 2025 Mar 11;12:1461319. doi: 10.3389/fsurg.2025.1461319 (PMC11933057; doi:10.3389/fsurg.2025.1461319)

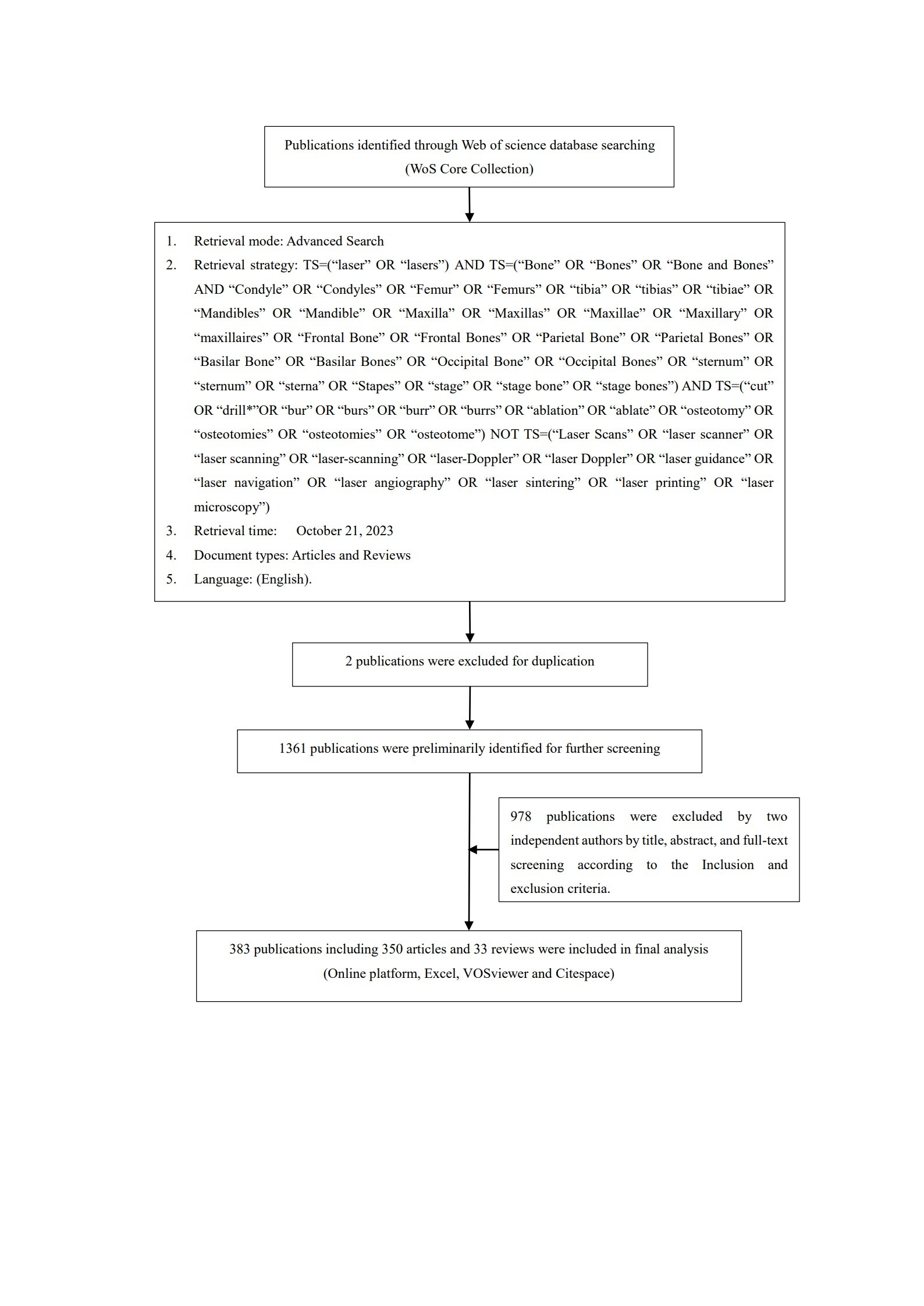

Supplement: Supplementary file 1 [file Datasheet1.zip › Supplementary material-figures/Figure 1.jpg]

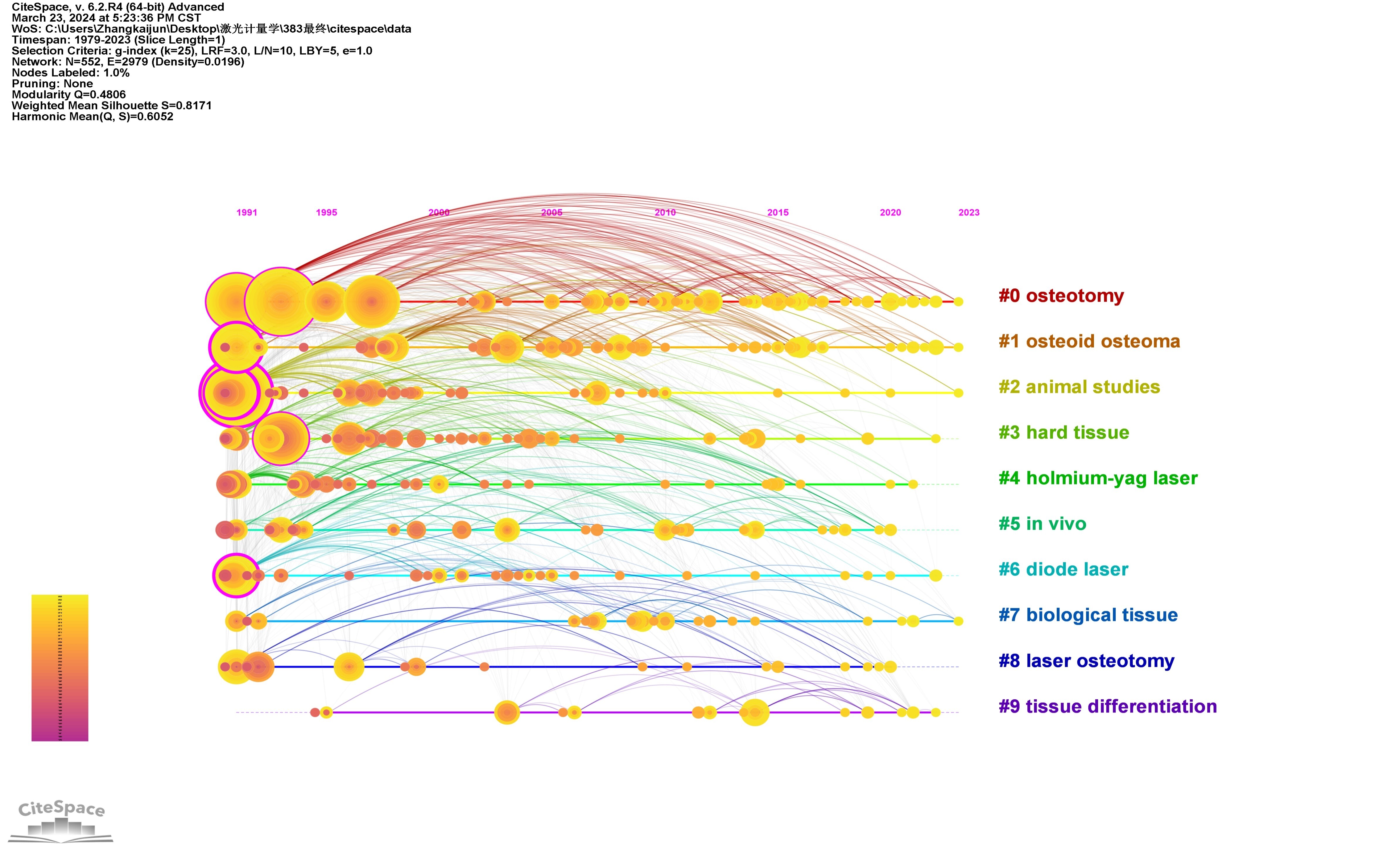

Supplement: Supplementary file 1 [file Datasheet1.zip › Supplementary material-figures/Figure 10.jpg]

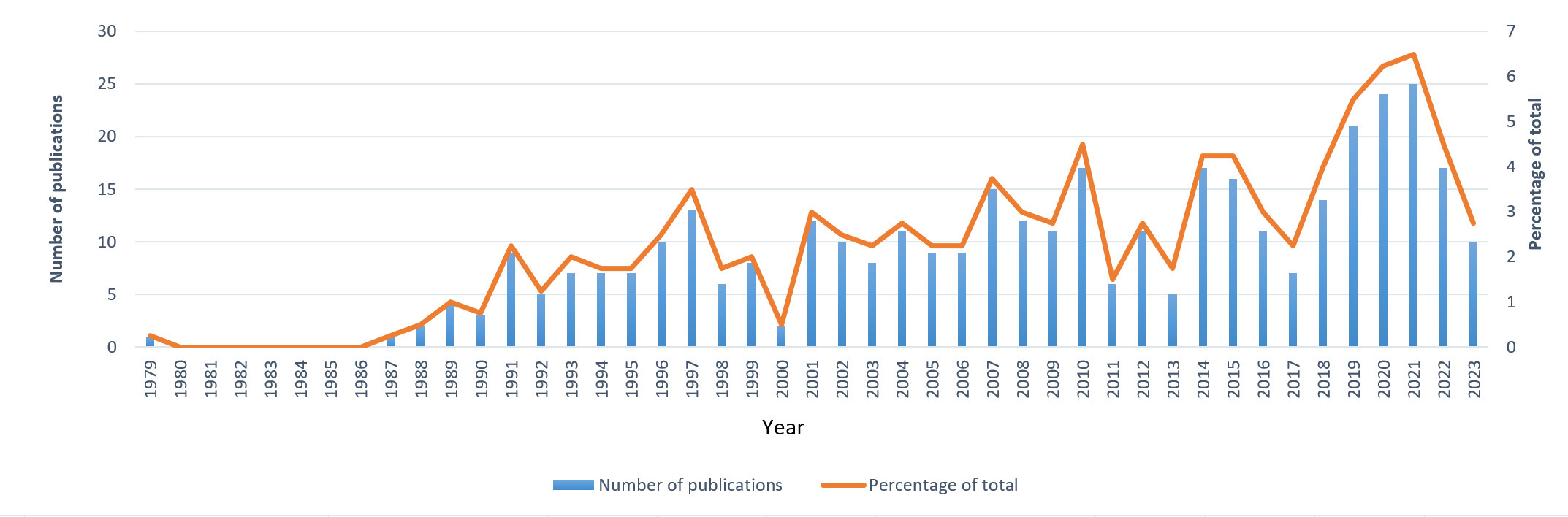

Supplement: Supplementary file 1 [file Datasheet1.zip › Supplementary material-figures/Figure 2 A.jpg]

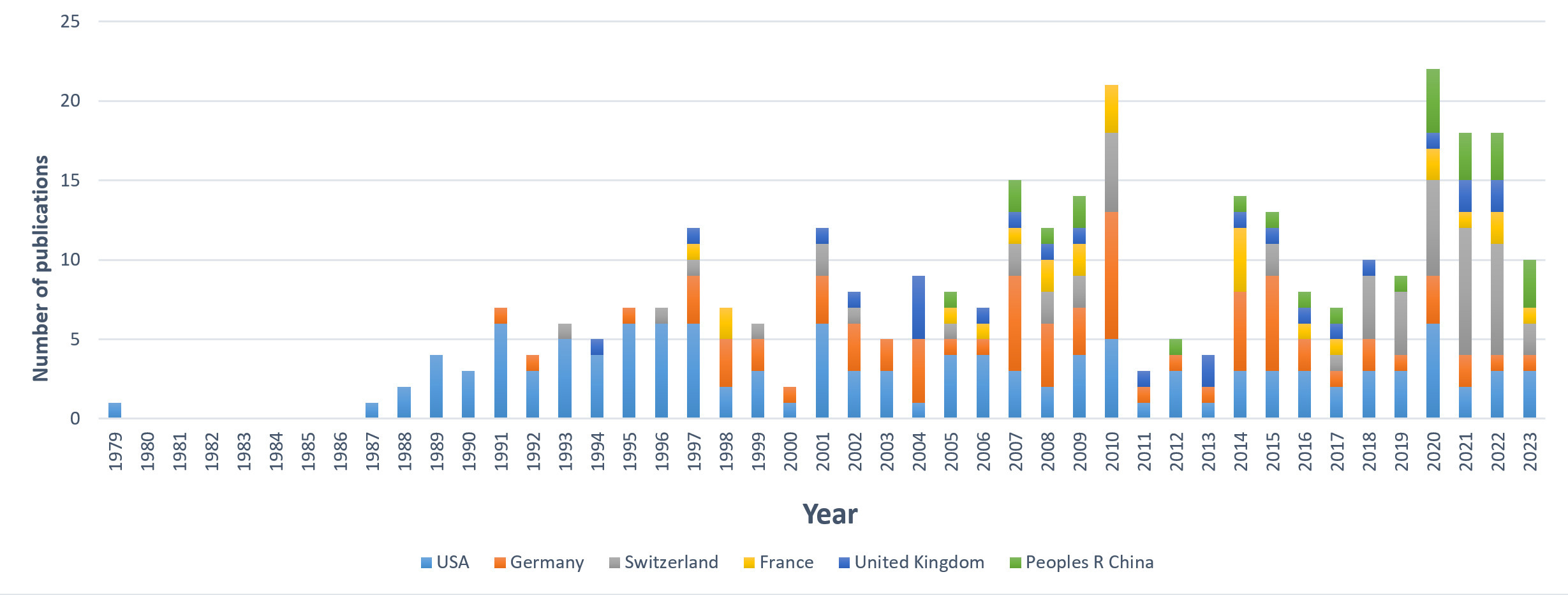

Supplement: Supplementary file 1 [file Datasheet1.zip › Supplementary material-figures/Figure 2 B.jpg]

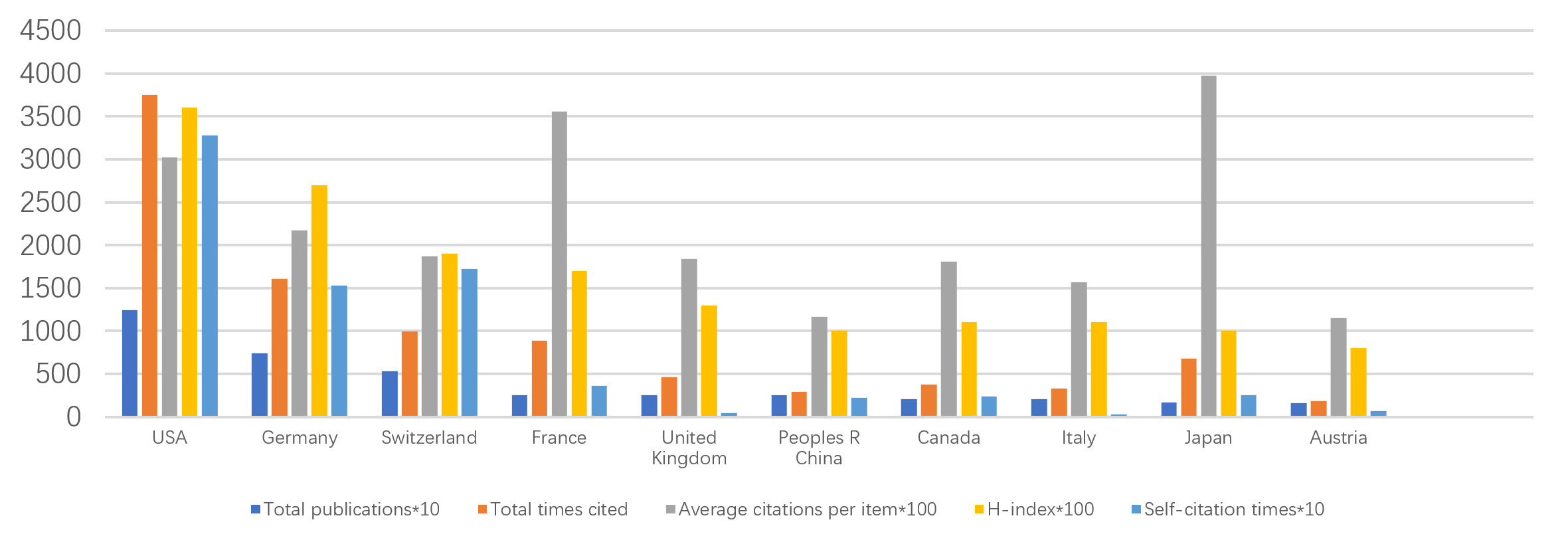

Supplement: Supplementary file 1 [file Datasheet1.zip › Supplementary material-figures/Figure 3 A.jpg]

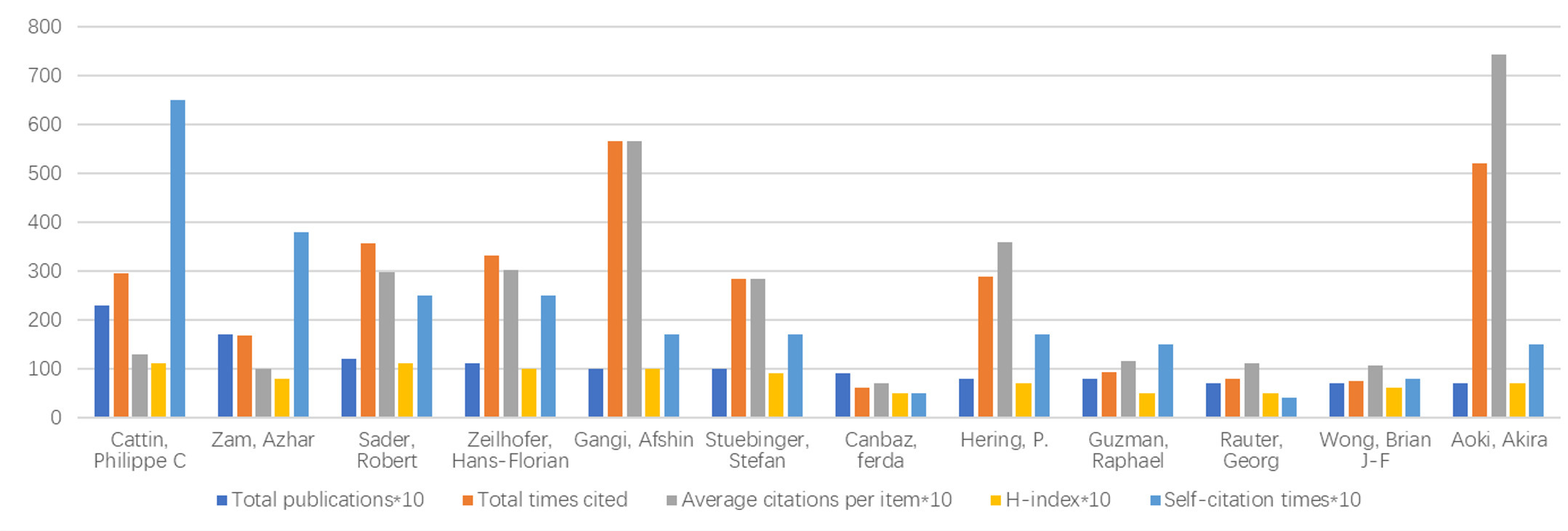

Supplement: Supplementary file 1 [file Datasheet1.zip › Supplementary material-figures/Figure 3 B.jpg]

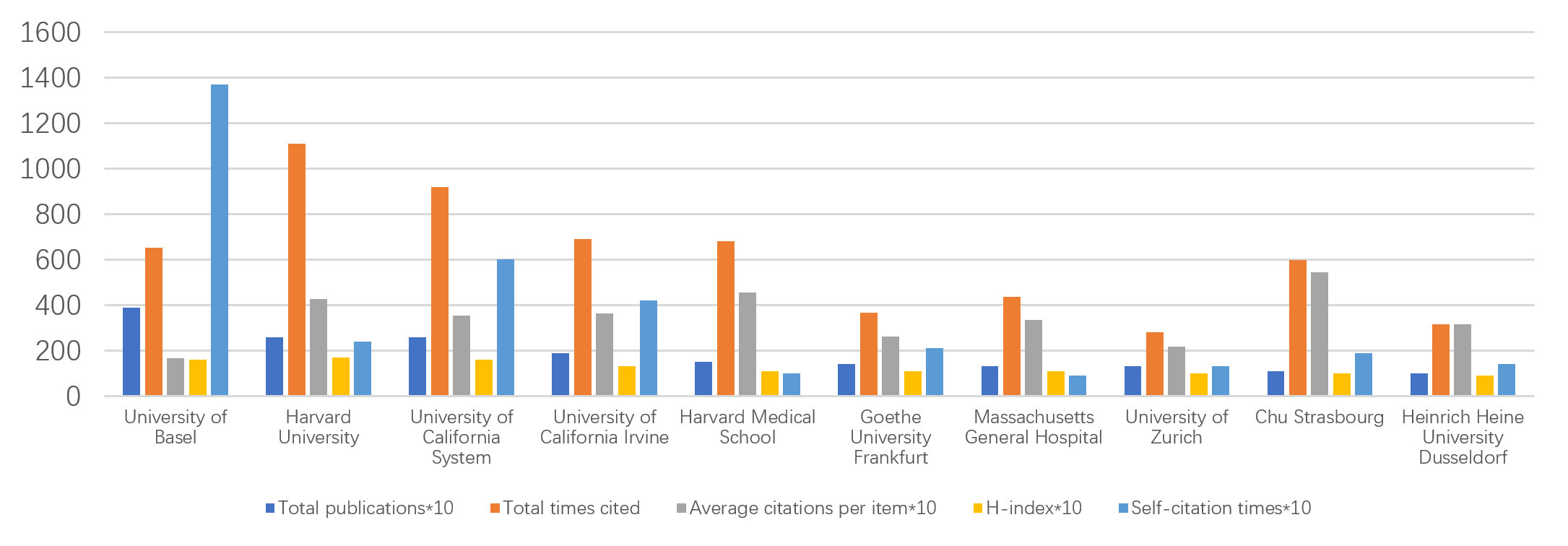

Supplement: Supplementary file 1 [file Datasheet1.zip › Supplementary material-figures/Figure 3 C.jpg]

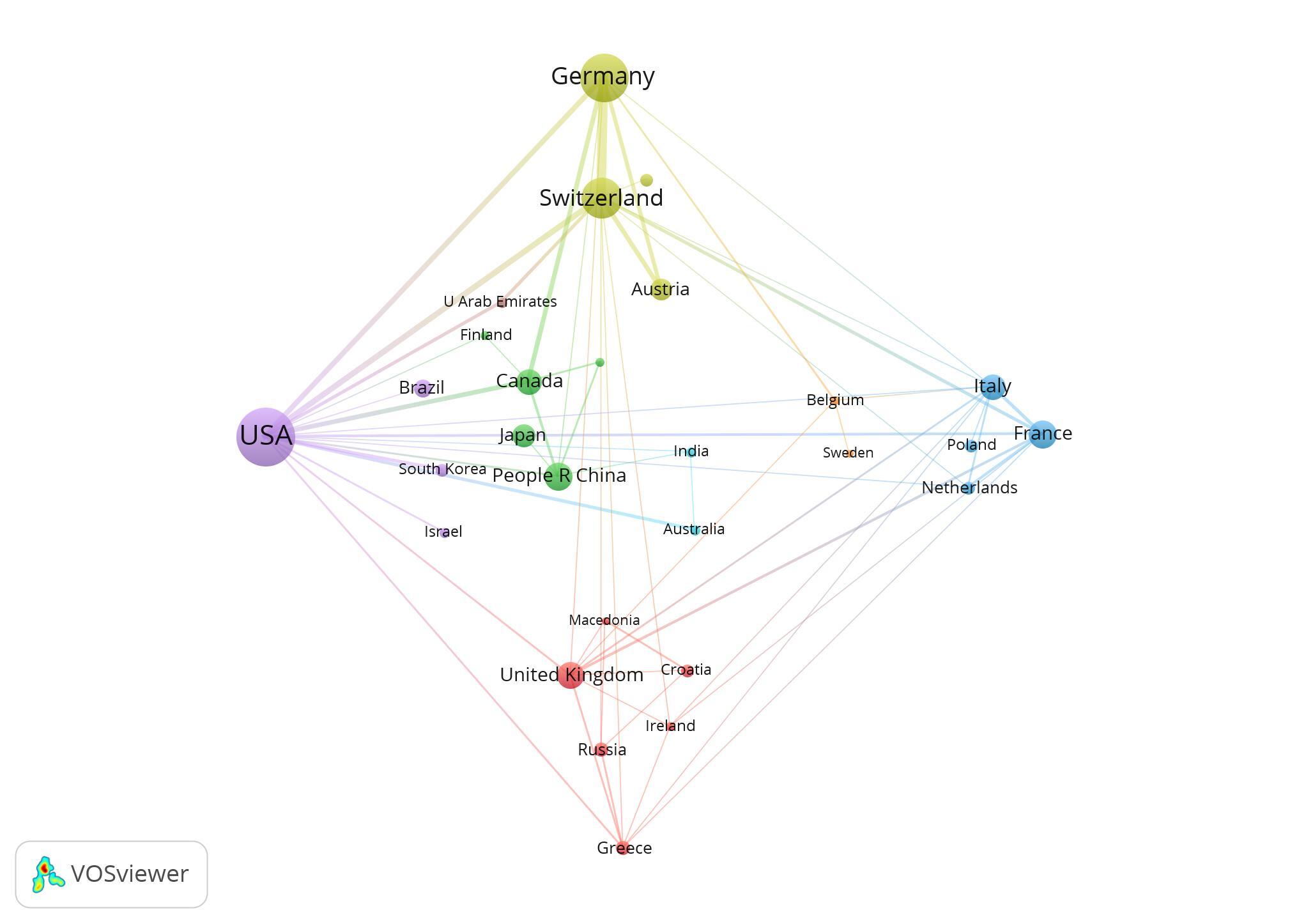

Supplement: Supplementary file 1 [file Datasheet1.zip › Supplementary material-figures/Figure 4 A.jpg]

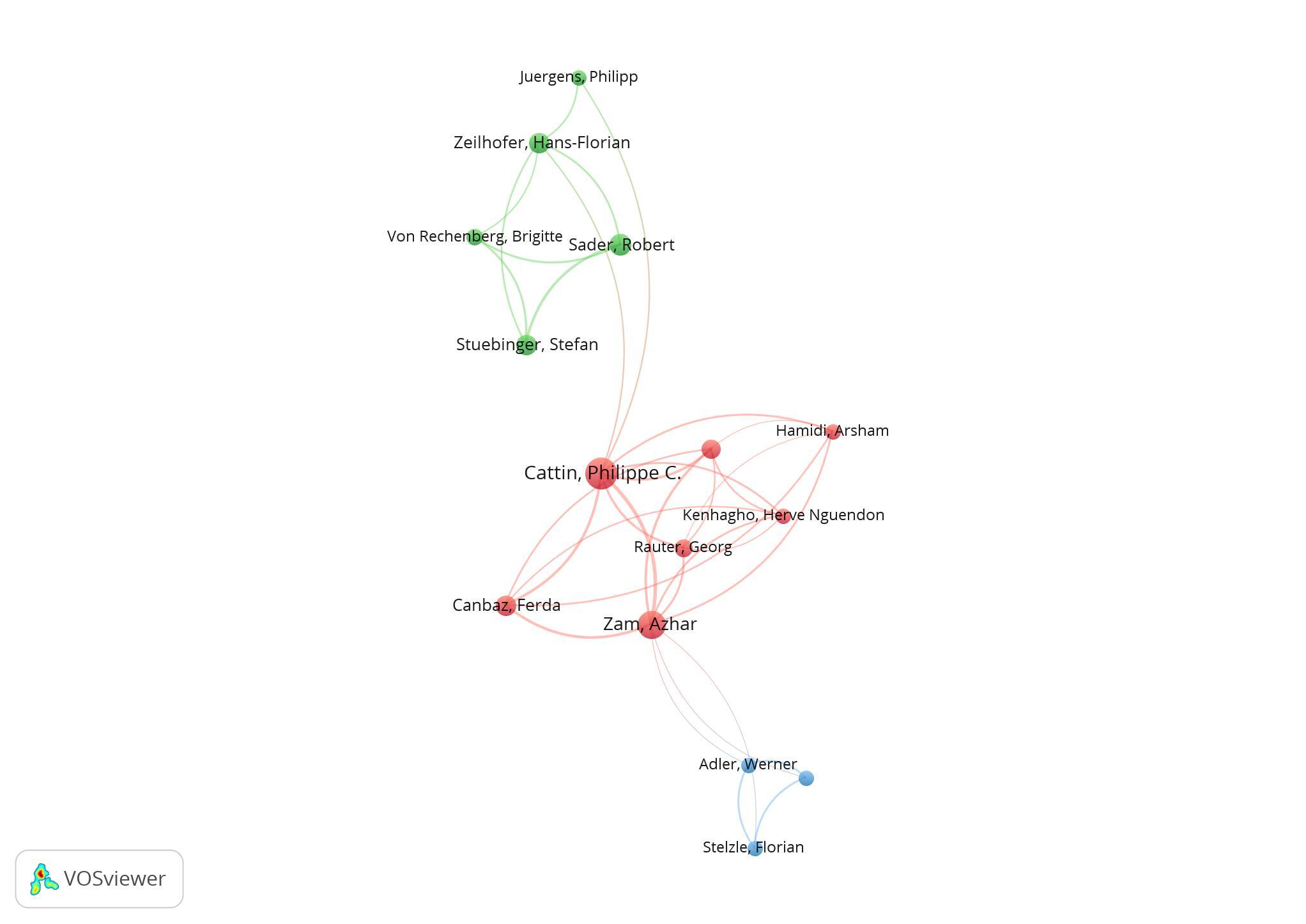

Supplement: Supplementary file 1 [file Datasheet1.zip › Supplementary material-figures/Figure 4 B.jpg]

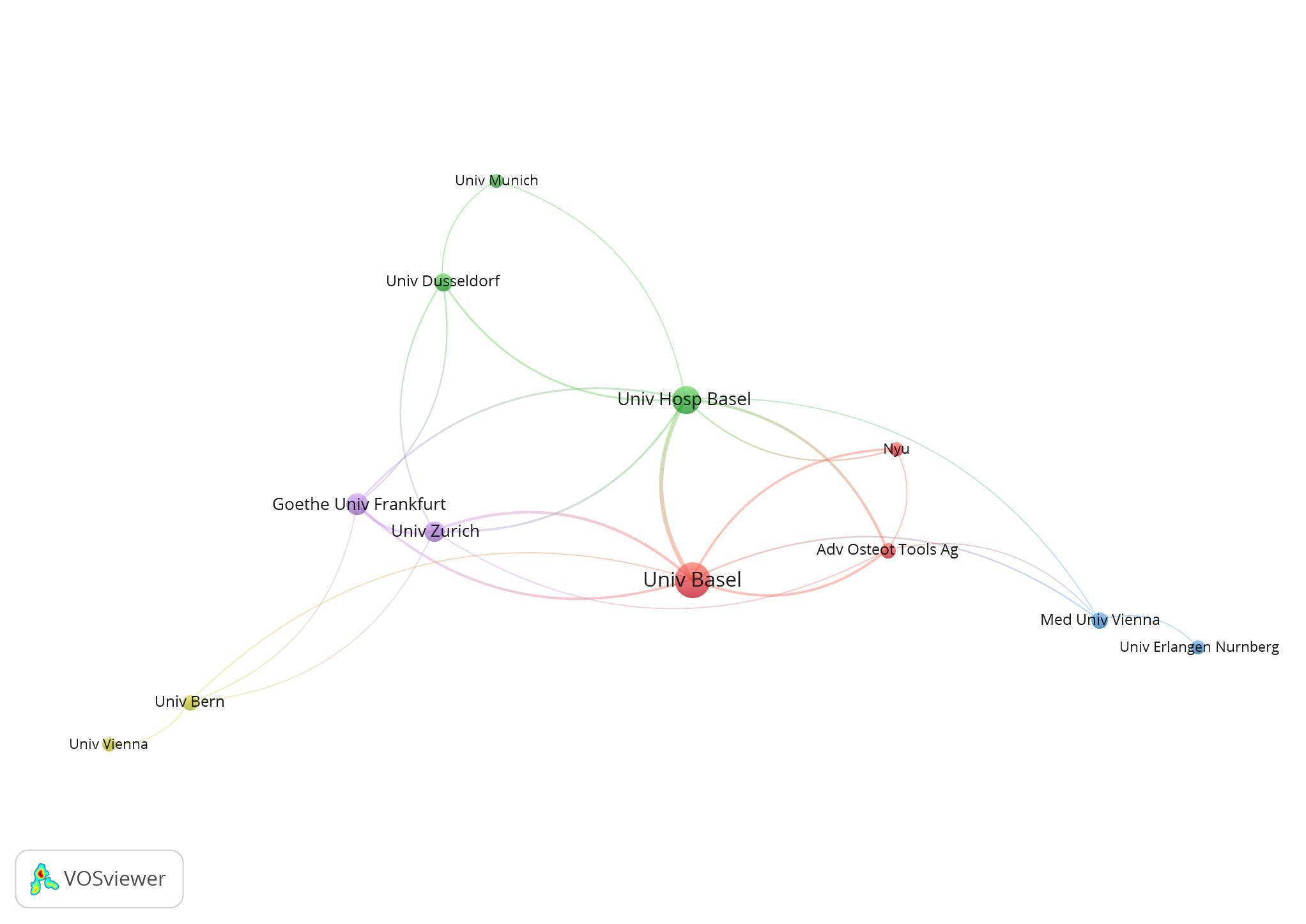

Supplement: Supplementary file 1 [file Datasheet1.zip › Supplementary material-figures/Figure 4 C.jpg]

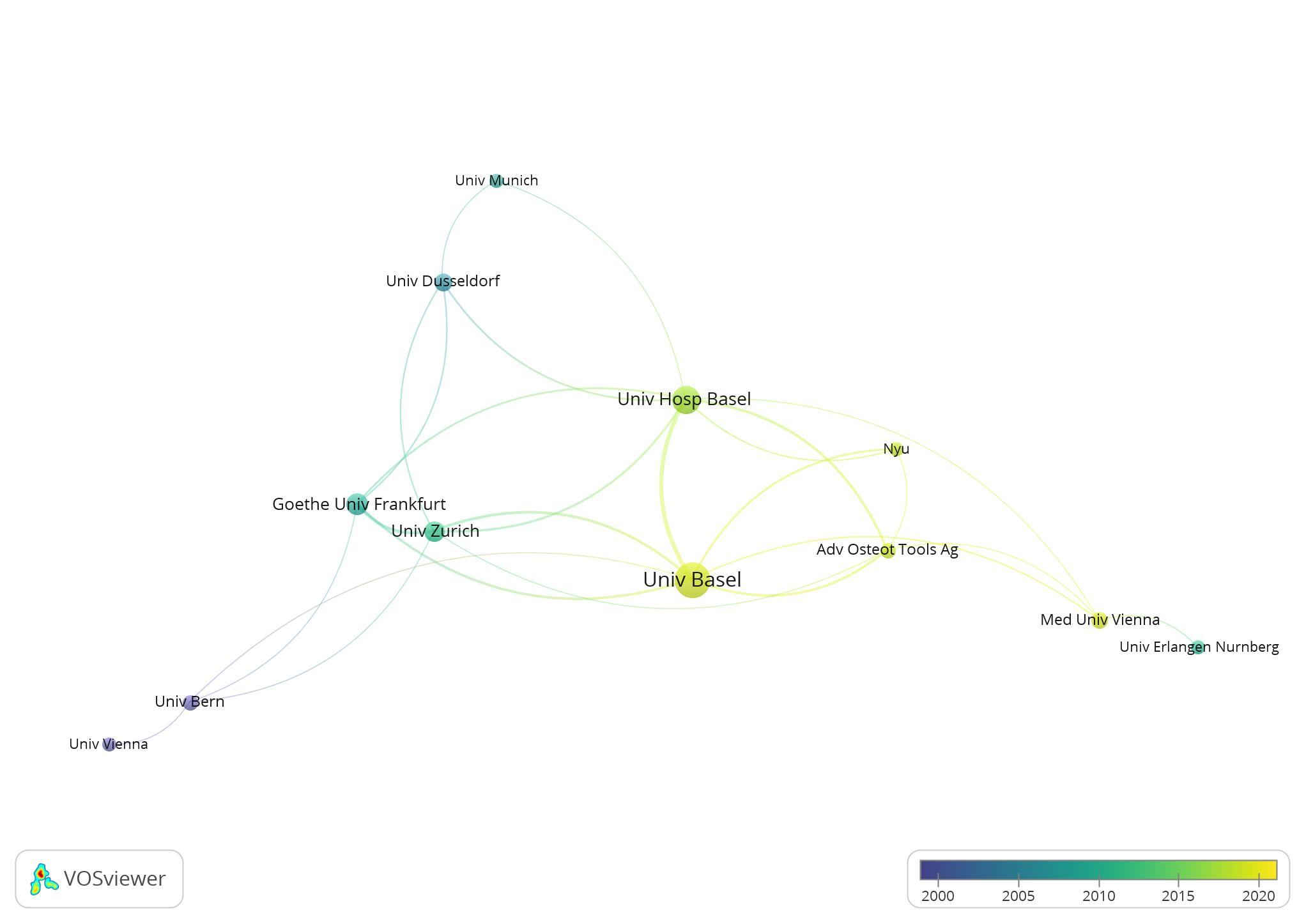

Supplement: Supplementary file 1 [file Datasheet1.zip › Supplementary material-figures/Figure 4 D.jpg]

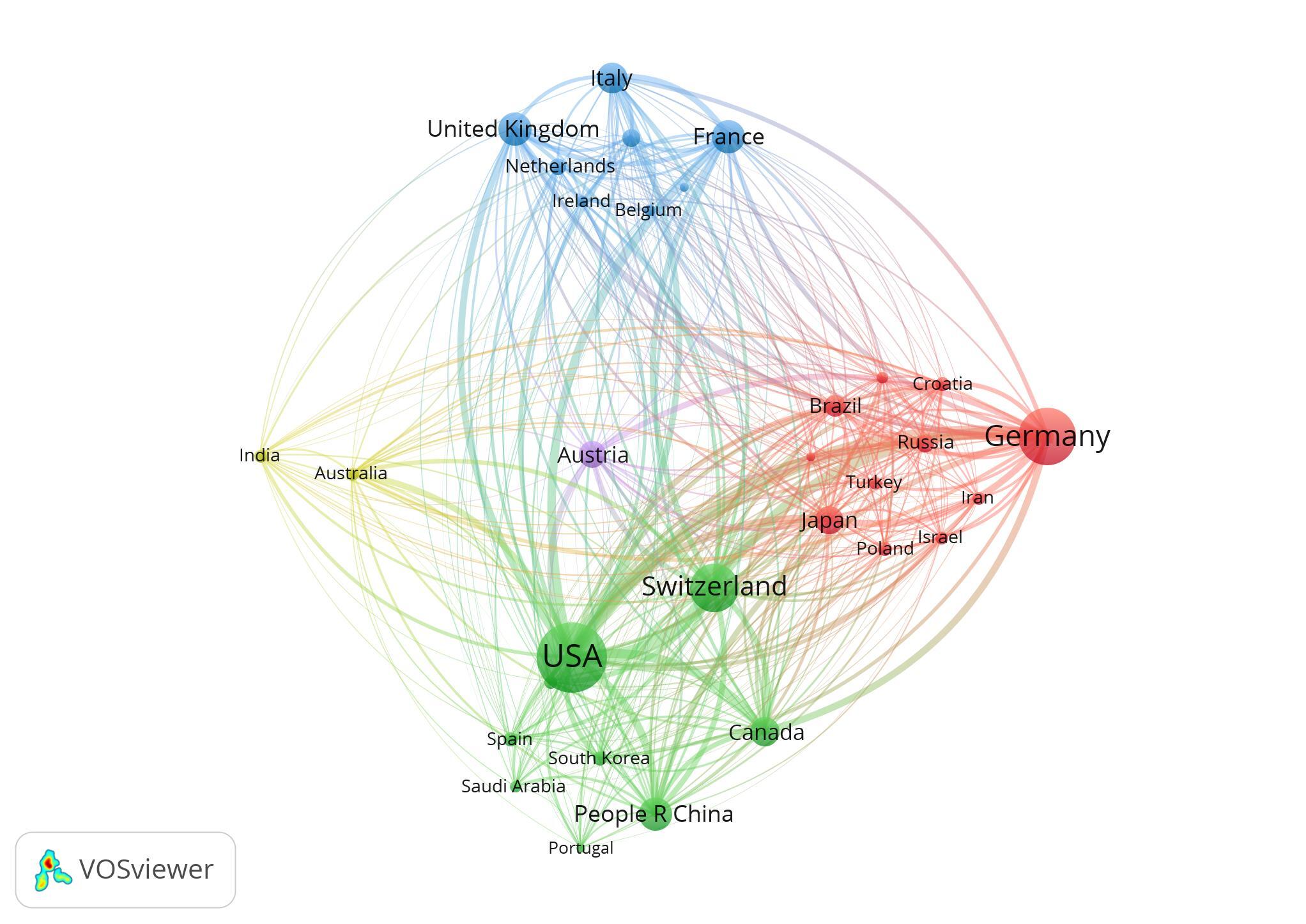

Supplement: Supplementary file 1 [file Datasheet1.zip › Supplementary material-figures/Figure 5.jpg]

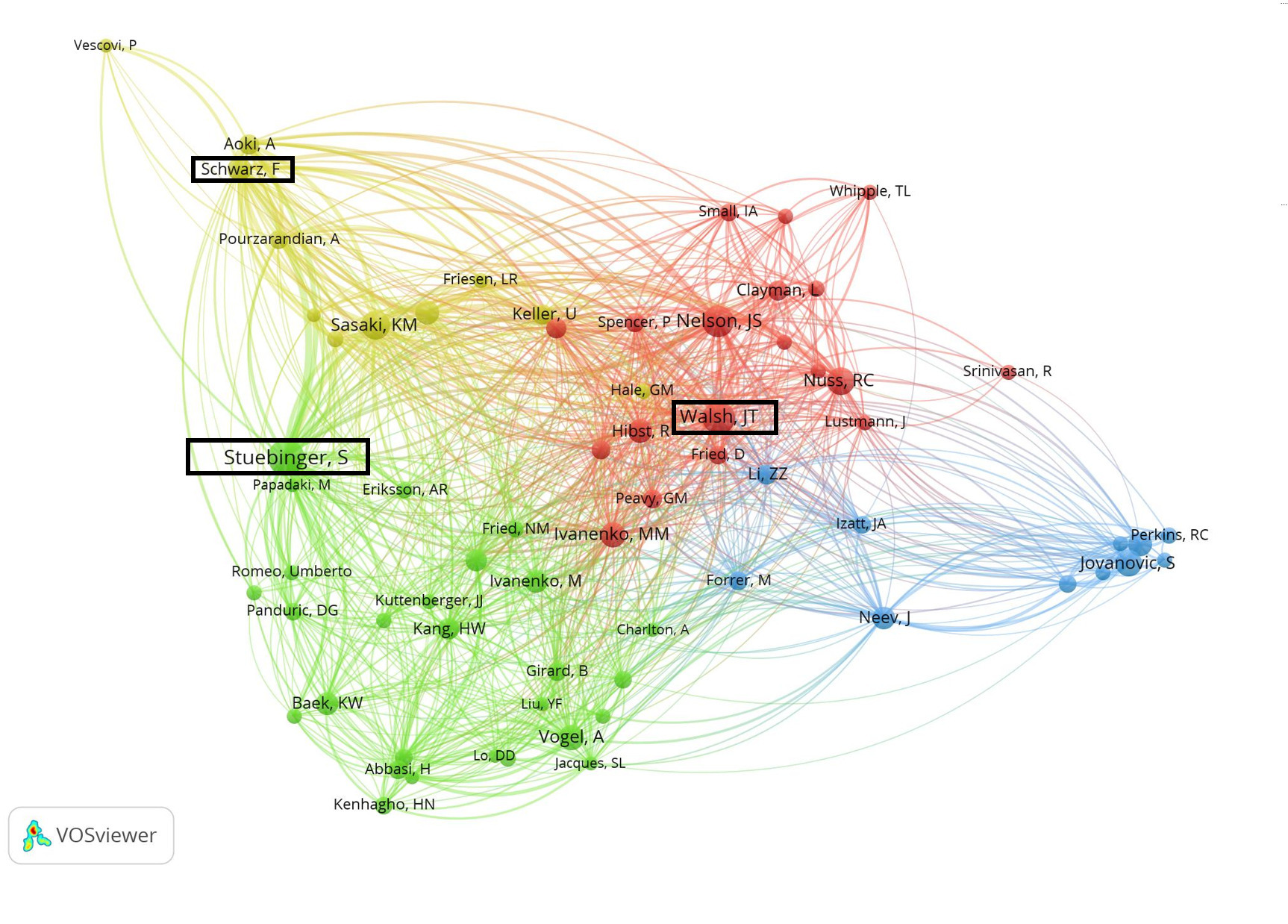

Supplement: Supplementary file 1 [file Datasheet1.zip › Supplementary material-figures/Figure 6 A.jpg]

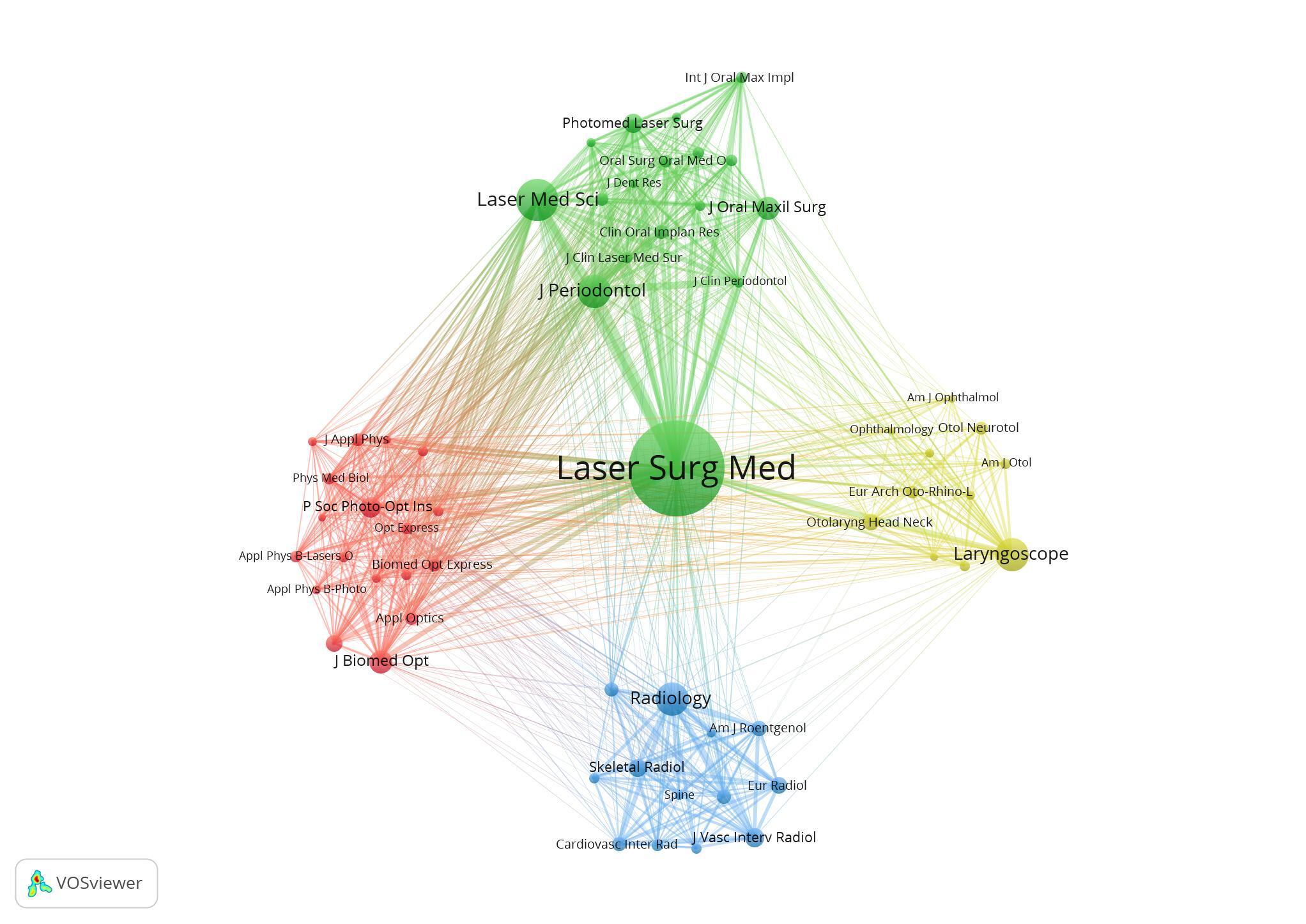

Supplement: Supplementary file 1 [file Datasheet1.zip › Supplementary material-figures/Figure 6 B.jpg]

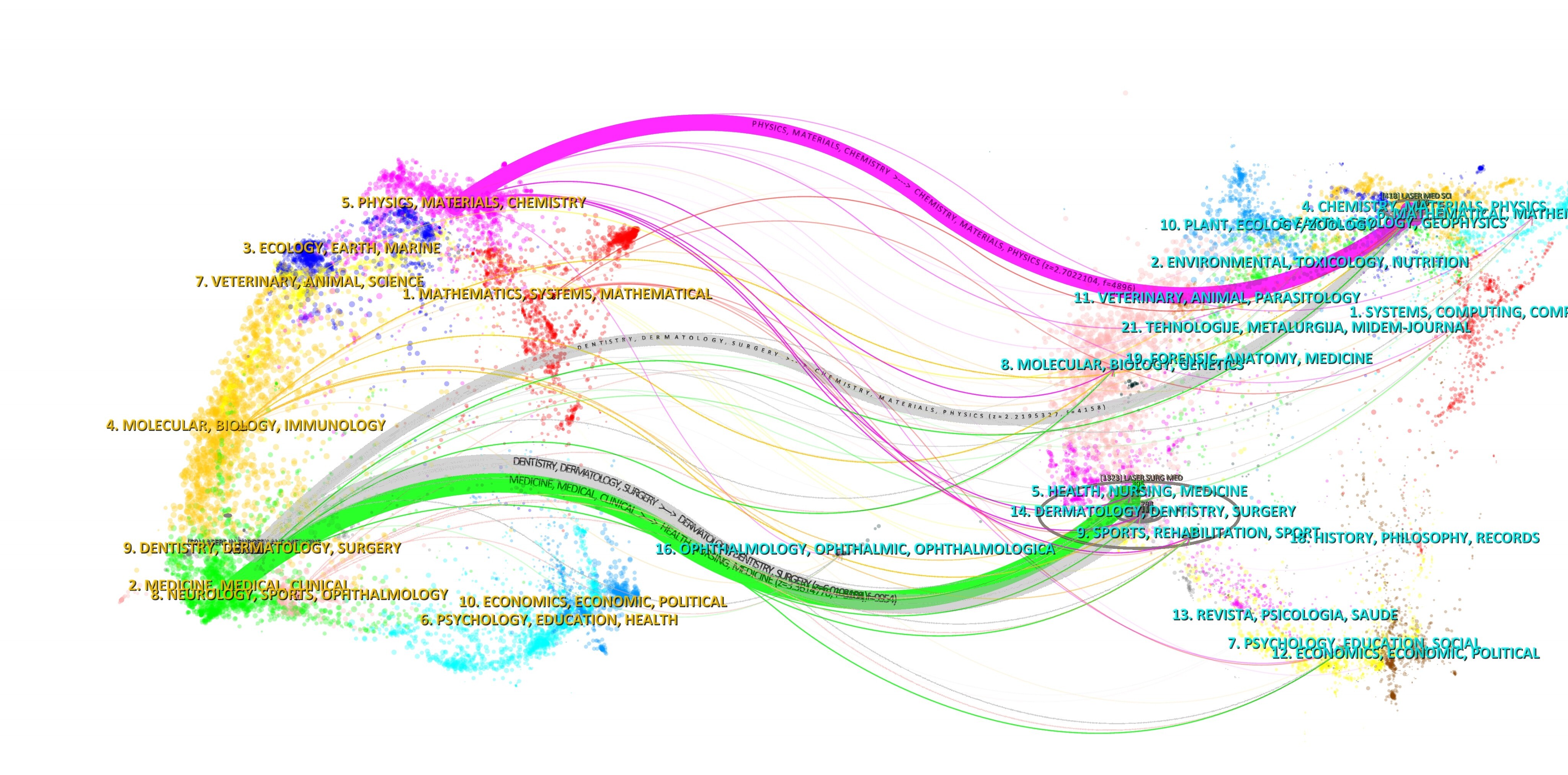

Supplement: Supplementary file 1 [file Datasheet1.zip › Supplementary material-figures/Figure 7.jpg]

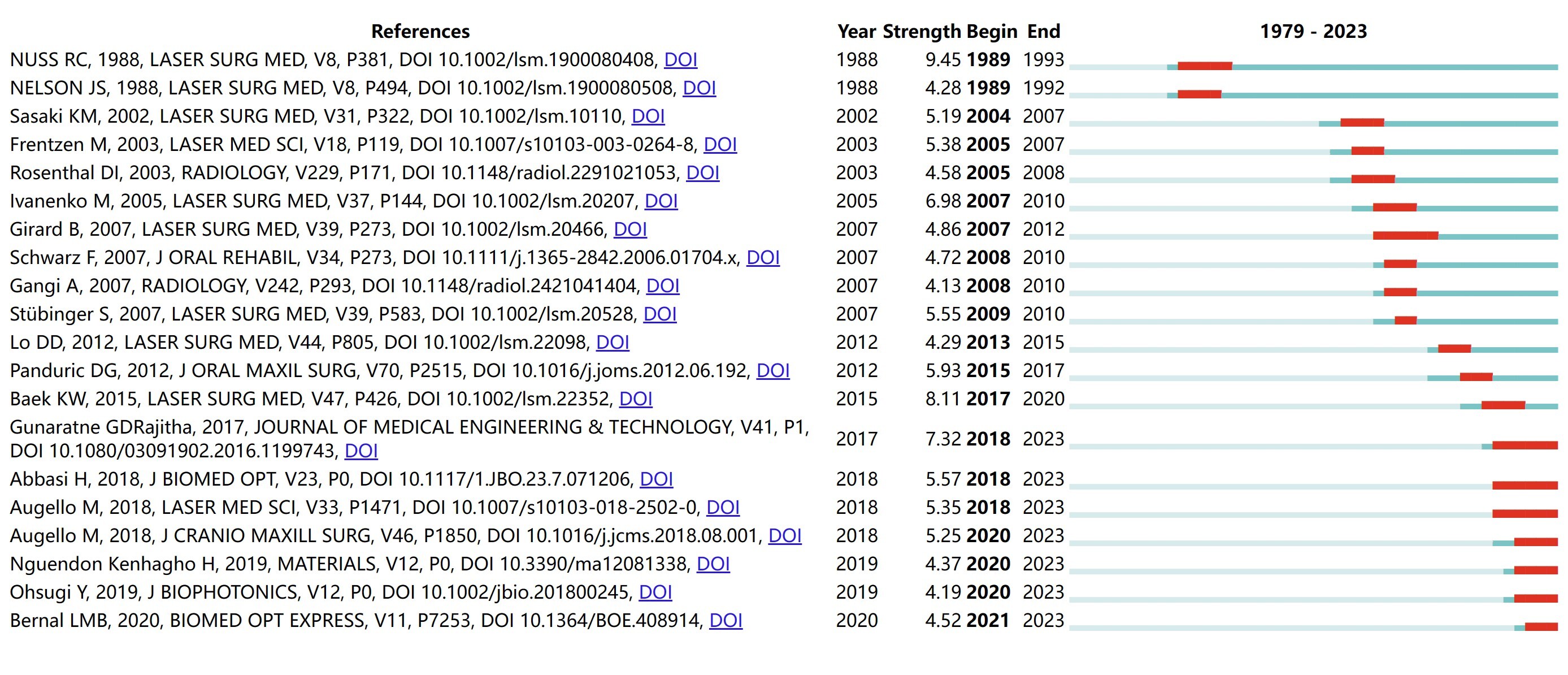

Supplement: Supplementary file 1 [file Datasheet1.zip › Supplementary material-figures/Figure 8.jpg]

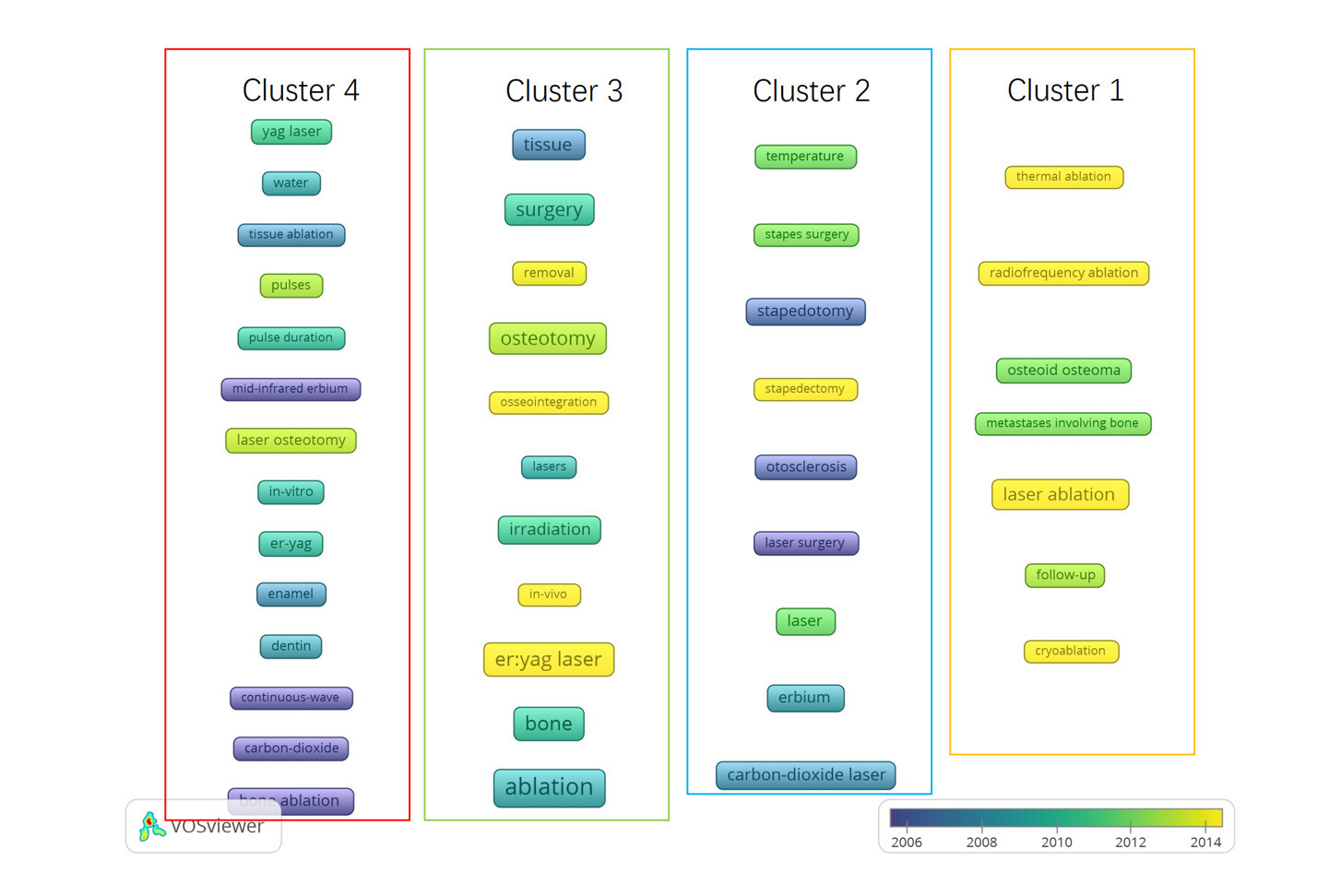

Supplement: Supplementary file 1 [file Datasheet1.zip › Supplementary material-figures/Figure 9.jpg]
